# Psychosocial Interventions and Support Groups for Siblings of Individuals with Neurodevelopmental Conditions: A Mixed Methods Systematic Review of Sibling Self-reported Mental Health and Wellbeing Outcomes

**DOI:** 10.1007/s10567-022-00413-4

**Published:** 2022-09-30

**Authors:** Brittany Wolff, Iliana Magiati, Rachel Roberts, Rachel Skoss, Emma J. Glasson

**Affiliations:** 1 School of Psychological Science, The University of Western Australia, Perth, Australia; 2 Telethon Kids Institute, Centre for Child Health Research, The University of Western Australia, Perth, Australia; 3 School of Psychology, The University of Adelaide, Adelaide, Australia; 4 Institute for Health Research, The University of Notre Dame Australia, Perth, Australia; 5 School of Population and Global Health, The University of Western, Perth, Australia; 6 Discipline of Psychiatry, Medical School, The University of Western Australia, Perth, WA Australia

**Keywords:** Neurodevelopment, Disability, Sibling, Mental health, Wellbeing, Intervention, Outcome

## Abstract

Siblings of persons with neurodevelopmental conditions (NDCs) have increased risk of poorer psychosocial functioning. This systematic review evaluated quantitative and qualitative evidence on sibling mental health and wellbeing outcomes following psychosocial interventions and the risk and protective factors associated with post-intervention outcomes. From 2025 identified studies published from 1991 to 2022 across ten databases, 24 studies were included. The largest immediate post-intervention improvements were in self-esteem, social wellbeing and knowledge of NDCs. The most sustained improvements in intervention groups at follow-up periods were in emotional and behavioural adjustment and NDC knowledge. There were positive, but small, differences in favour of the intervention groups on knowledge of NDCs, self-esteem, coping and the sibling relationship as compared to waitlist control groups. Psychosocial interventions for siblings are heterogeneous, and more data, including consideration of unique family circumstances, are needed to improve reporting and replicability, to measure effectiveness and tailor necessary supports.

## Introduction

In the Diagnostic and Statistical Manual for Mental Disorders, Fifth Edition (DSM-5; American Psychiatric Association, 2013) individuals with neurodevelopmental disorders (hereafter neurodevelopmental conditions; NDCs) include those with intellectual disabilities, autism spectrum disorder, attention-deficit/hyperactivity disorder (ADHD)^1^ and specific learning, communication and motor disorders (American Psychiatric Association, 2013). NDCs are a group of conditions with onset in the developmental period, characterised by developmental difficulties that produce impairments of personal, social, academic and/or occupational functioning. Many siblings of individuals with NDCs face unique individual and family challenges associated with their sibling’s condition (Hayden et al., 2019; Marquis et al., 2019).^2^ Meta-analyses indicate that, overall as a group, siblings of persons with NDCs experience a small yet significant overall negative impact on psychological and neurocognitive development and functioning compared to siblings without disabilities (Rossiter & Sharpe, 2001; Vermaes et al., 2012), with negative impact greatest for siblings of autistic children (Shivers et al., 2019). Siblings may experience increased risk of stress, anxiety, depression and related adjustment difficulties (Sharpe & Rossiter, 2002; Shivers et al., 2019; Vermaes et al., 2012), overall poorer mental health and wellbeing (Giallo et al., 2012; Marquis et al., 2019) and lower self-concept and more behaviour problems (Fisman et al., 2000) than comparison groups of siblings of individuals without NDCs, the general population or normative samples (Lin et al., 2021; Marquis et al., 2020).

Reviews on outcome studies of siblings of individuals with heterogeneous disabilities including mixed groups of participants with chronic physical or psychiatric conditions as well as NDCs report both negative and positive effects of having a sibling with a disability (Knecht et al., 2015; Mandleco & Webb, 2015; Marquis et al., 2020; McKenzie Smith et al., 2018), while qualitative syntheses emphasise positive effects more prominently, finding that siblings may experience increased empathy, maturity, understanding, patience and love (Cridland et al., 2014; Lamsal & Ungar, 2021; Leedham et al., 2020; Lummer-Aikey & Goldstein, 2021; Watson et al., 2021). Previous sibling studies have identified a range of individual, family and structural level risk and resilience-promoting factors that may influence sibling wellbeing outcomes. Examples include low socioeconomic status, single parent families, belonging to a non-Western or non-Caucasian minority group (Marquis et al., 2019; Viswanathan et al., 2021), parental mental health conditions or stress (Chen et al., 2019), higher NDC severity, more externalizing behavioural problems (Pollard et al., 2013) and reduced access to services (Pavlopoulou & Dimitriou, 2020). Conversely, protective factors include higher perceived social support (Koukouriki et al., 2021), more effective family communication and cohesion (Okashah et al., 2015; Pavlopoulou & Dimitriou, 2019), better family knowledge of NDCs (Hastings & Petalas, 2014; Haukeland et al., 2020) and the quality of the sibling relationship (Pollard et al., 2013), amongst others. These factors may influence both sibling outcomes more broadly as well as influence the extent to which some siblings may benefit more from interventions and supports as compared to others (Tudor & Lerner, 2015).

Five previous systematic reviews have to date reported on psychosocial and educational interventions for heterogeneous groups of siblings of children with NDCs or chronic illnesses, representing another type of long-term condition that may impact sibling experiences (Hartling et al., 2014; Kirchhofer et al., 2022; McKenzie Smith et al., 2018; Thomas et al., 2016; Tudor & Lerner, 2015). In Hartling et al. (2014), 14 studies published between 1989 and 2007 delivered psycho-educational interventions (coping and social skills, improving knowledge of illnesses) to siblings of individuals with chronic illness and medical conditions (e.g. cancer, physical disabilities) or with other conditions (e.g. intellectual disability, unspecified NDCs) with a median sample size of 24, siblings aged 6–17 years and siblings with NDCs and other physical/medical/chronic conditions aged 4–18 years; thus, this review included heterogeneous samples of siblings with other chronic illnesses. Overall, small pre–post-intervention improvements were reported by either siblings or parents in 10 of the 14 studies for outcomes including anxiety, depression, self-esteem, self-concept, sibling relationship, increased knowledge of illness, mood and positive attitudes towards their sibling.

Tudor and Lerner (2015) reviewed 16 studies published between 1985 and 2010 evaluating psychosocial and educational support programmes for siblings of children with NDCs, with sample sizes ranging from three to 252 and siblings aged 6–12 years; on average 57% of the siblings were older than their siblings with NDCs (who were aged 1–19 years). The review found positive significant or non-significant caregiver reported improvements post-intervention in 10 of the 16 studies (five of which had a comparison group), including improved social support, self-esteem, knowledge about disability, sibling relationships, emotional and behavioural adjustment and sibling enjoyment. The limitations of this review were the exclusion of qualitative studies and that siblings’ outcomes were reported solely by parents. Thomas et al. (2016) reviewed eight psychosocial intervention studies for 4–16 year old siblings of autistic individuals only (who were 3–17 years old), six of which used only parent−report measures. They found that only two outcomes, sibling relationship quality and understanding about autism, were improved post-intervention.

McKenzie Smith et al. (2018) systematic review and meta-analysis included 17 studies published between 1990 and 2015 on interventions for siblings (aged 6–15 years) of 3–17-year-old children with physical/medical conditions (e.g. cancer, cystic fibrosis), NDCs and mental health diagnoses. An overall small significant positive effect of interventions was found for sibling knowledge of NDC and parent-reported sibling behaviour problems. A limitation of this review was the restricted age range and combining of siblings of individuals with different conditions.

Finally, Kirchhofer et al. (2022) comprehensively examined social support for siblings of children with NDCs across 13 cross-sectional and two intervention studies, with a total sample of 1312 participant siblings aged 4–18 years. The review found a strong negative relationship in one study between social support and a range of mental health problems (Phillips, 1999). However, this review did not specifically focus on mental health outcomes and interventions, did not evaluate the mechanisms behind how the interventions may have improved social support and wellbeing and included parent-reported outcomes.

Across the five reviews summarised above, the interventions/support programmes were from a range of service providers (school, education, clinical), often implemented in manualised group session format with group sizes ranging from three to 17 participants. The sessions ranged from 1 to 2 h in length and ran for 6–10 weeks on a weekly basis. Most programmes focussed on psychoeducation and employed group discussions, disability-related activities, recreational games, social skills training and family-based cognitive−behavioural interventions (Hartling et al., 2014; Kirchhofer et al., 2022; McKenzie Smith et al., 2018; Thomas et al., 2016; Tudor & Lerner, 2015). However, it remains unclear which specific intervention components or characteristics contribute most to improving sibling outcomes.

### The Present Review: Rationale, Aims and Research Questions

No systematic review to date has identified and reviewed the risk and resilience-promoting factors characterising siblings who may benefit more than others from interventions in the included studies. Further, siblings’ qualitative experiences have not been systematically considered and no prior review has incorporated sibling community consultation. None of the reviews to date specifically focussed on siblings’ *self-reported* mental health and/or wellbeing outcomes following interventions. Self-report is integral to designing effective and tailored interventions according to siblings’ qualitative and unique experiences (Houtzager et al., 2005), particularly as parents have been found to both under- and over-report on siblings’ wellbeing and emotional adjustment, leading to reporting bias and overlooked sibling needs (McKenzie Smith et al., 2018; Rankin et al., 2017).

Therefore, the present review aimed to address the above limitations and conduct an up-to-date systematic review of intervention and support group studies of siblings of individuals with NDCs, focussing on self-reported outcomes, including both quantitative and qualitative studies and incorporating a sibling community consultation.

The following research questions were explored:

1. What are the quantitative outcomes of psychosocial interventions on siblings’ self-reported mental health/wellbeing?
2. What are the siblings’ qualitative experiences and perceptions of the benefits or detriments of these interventions?
3. What are siblings’ pre-intervention risk and resilience-promoting (protective) factors identified in the intervention studies associated with better or worse sibling outcomes post-intervention?

## Methods

This systematic review was pre-registered on Prospero, CRD42021264744. Our reporting was guided by the Preferred Reporting Items for Systematic Review and Meta-Analysis (PRISMA) Statement (Page et al., 2021).

### Eligibility Criteria

#### Participants

To be included in the present review, studies needed to report on participants who were siblings (children, adolescents or emerging adults) of individuals with NDCs, with a mean age for the sibling intervention group falling between 4 and 29 years. This age range was chosen in order to focus on children, adolescents *and* emerging adults, as many studies to date have either focussed on ‘infant siblings’ or ‘high risk’ sibling cohorts from birth to age four (Ozonoff et al., 2011); or on adult sibling relationships in the context of general disabilities (e.g. Rossetti & Hall, 2015). Childhood and adolescence are key developmental periods and emerging adulthood (encompassing ages 18–29) has distinctive demographic, social and psychological features (Arnett, 2010) and remains understudied. Studies not including sibling outcomes as the main target of intervention were excluded (e.g. sibling-mediated interventions for the NDC sibling).

#### Exposure

To be included in this review, studies needed to report on psychosocial interventions, which were defined as interventions focussing on the psychological, behavioural or social factors associated with the sibling rather than biological factors, such as pharmacotherapy. Studies reporting on any intervention or support programme delivered via any psychosocially focussed approach and of any format (i.e. manualised or not; group setting) were included, provided the aim was to improve sibling psychological functioning and/or wellbeing.

#### Phenomena of Interest

To be included in this review, studies needed to include participants who had a sibling (of any age) with an NDC as defined in the DSM-5 (American Psychiatric Association, 2013; see section “Introduction”). In studies including various NDCs or chronic illnesses in the disabled sibling, at least 50% of the overall sample of disabled siblings must have had a diagnosed or parent-reported NDC to be included. Any intervention study with three or more sibling participants was included, due to the known difficulty in recruiting siblings of individuals with NDCs (Vermaes et al., 2012).

#### Comparison

Due to the nature of many interventions being pilot programs, studies were not required to include a comparison group.

#### Outcomes

The primary outcomes were sibling mental health/wellbeing as measured by at least one sibling self-report measure. This review considered categorical (i.e. sibling diagnosed/not diagnosed with a mental health condition using standardised classification systems including the DSM-5 and ICD-10) and dimensional measures of mental health (i.e. ratings in checklists). Wellbeing was conceptualised as a subjective, dynamic construct covering mental, physical, emotional and environmental states (Kiefer, 2008); or subjective interpretations of quality of life (Moyson & Roeyers, 2011). Studies without a primary focus on sibling mental health or wellbeing were excluded. Studies could include parent report provided at least one outcome was sibling self-report.

#### Study Type

Studies could employ quantitative only, qualitative only or mixed methods designs (a combination of quantitative and qualitative methods). Both cross-sectional (i.e. intervention designs with only one data collection point during or after the intervention) and follow-up studies (pre–post design and longer follow-up designs) were included.

#### Publication Type and Date

Other systematic reviews and grey literature (e.g. dissertations) were excluded. Only published records in English, French or Danish were included. Dates of coverage included all available records from conception of the database until the final search date of 24 July, 2022.

### Information Sources

Before commencing, other systematic review databases (Cochrane Library, Prospero, JBI EBP, DoPHER) were searched for similar registered reviews and no searches produced systematic review protocols involving the key terms for this review. Databases searched included: Ovid MEDLINE, Embase, CINAHL, PsycINFO, PsycARTICLES, Pubmed, ProQuest, Web of Science, Scopus and Google Scholar.

### Search Strategy

A pilot search was conducted. Search strings were identified and run which maximised sensitivity and minimised the absolute difference between sensitivity and specificity. We allowed higher sensitivity than specificity to ensure all/most relevant records were found and the best search string optimisation achieved 91.1% sensitivity and 83.2% specificity, with a precision of 5.1%. The search was limited to titles and abstracts. The search terms included database specific controlled vocabulary, field codes, operators, relevant keywords and subject headings to identify the population of interest (siblings), the exposure (psychosocial interventions) and the outcome (mental health or wellbeing). A comprehensive list of search terms to encompass mental health outcomes following interventions for siblings of persons with NDCs were included and these terms are presented in full in Table 1 of the Appendix. Search terms in each category were combined using the Boolean operator ‘OR’; search categories were combined using the Boolean operator ‘AND’. The final search strategy and the specific search terms are presented in Table 1 (Appendix).

### Selection Process

The first author (BW) screened all identified records by reading titles and abstracts, excluding abstracts clearly not meeting the eligibility criteria and then reading full texts after this initial screening. The first author (BW) then decided on final records for inclusion based on the meeting all of the above eligibility criteria; any studies with questionable eligibility were screened by the last author (EG) and discussed to reach an agreement on inclusion. Further, a random 20% of full texts identified were screened by the last author (EG) and inter-rater agreement was very good (85%). Any disagreements were discussed and resolved. The final list of records was read by the last author (EG), following which forwards and backwards citation searching was conducted for all eligible articles, leading to a further six articles included. These six studies met all eligibility criteria described above and were agreed upon by the first and last authors.

**Fig. 1:**
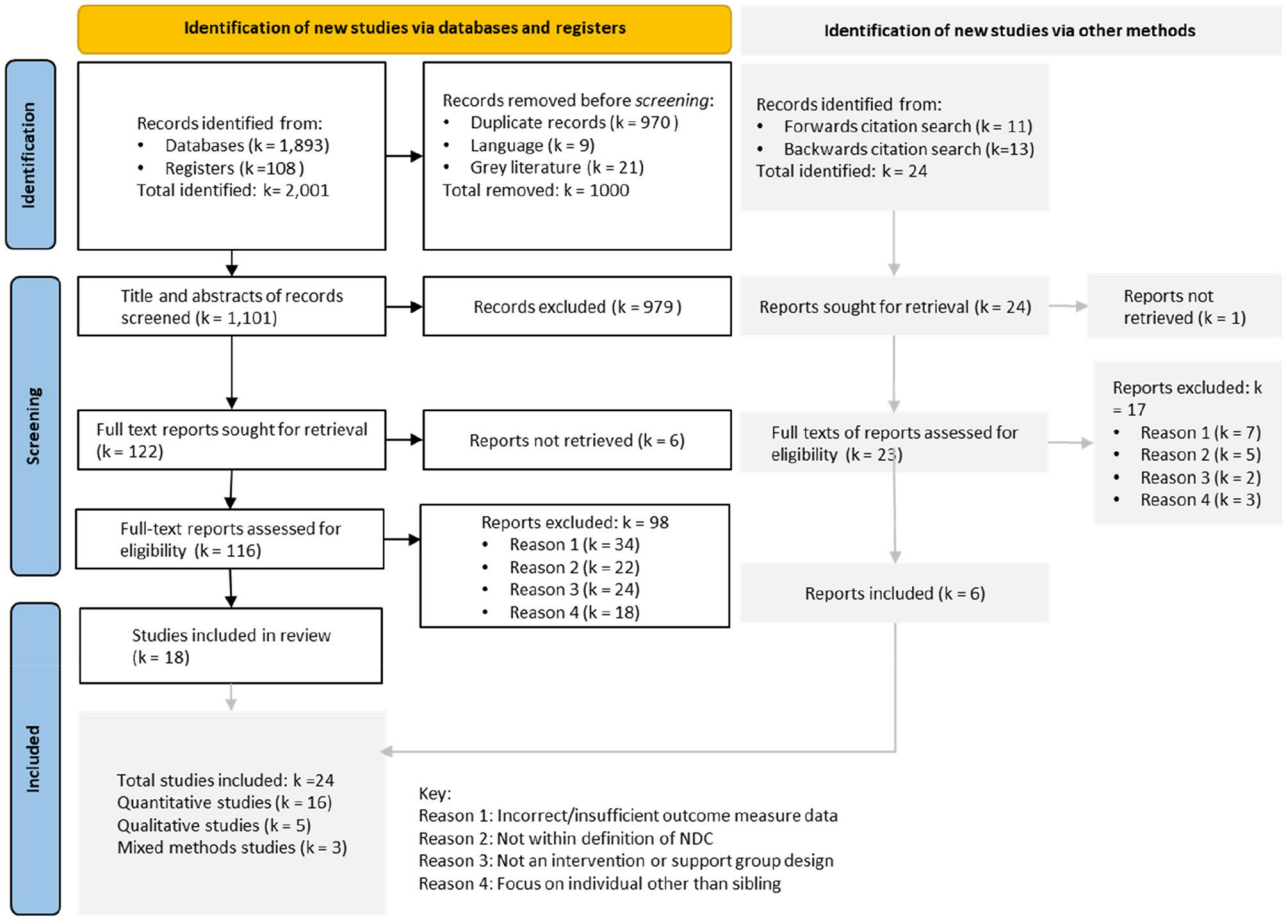
PRISMA flowchart depicting selection of studies (Page et al., 2021)

The most common reasons for exclusion of articles were: incorrect or insufficient intervention/programme outcome data; the sibling disability not falling within our definition of an NDC; the study not being an intervention or support group design; or the study focussing on an individual other than the sibling (e.g. the parents or a sibling-mediated intervention designed for the sibling with an NDC; see Fig. 1).

### Data Collection Process

A standardised form used to extract data from the eligible studies was piloted on a validation dataset. Search results were exported to EndNote citation manager and duplicates were removed. Full-text papers were read by two study investigators independently (BW, EG). No automaton tools were used.

### Data Items

The first author (BW) extracted all data items (see Tables 2 and 3), which were then cross-checked by the last author (EG).

### Effect Measures

A meta-analysis of effect sizes was not carried out due to the large heterogeneity in measures used, intervention designs and sibling sample characteristics. The body of evidence across sibling-reported mental health/wellbeing outcomes were coded as overall positive (a beneficial effect of the intervention), overall negative (a negative effect of the intervention) or neutral (no significant difference pre–post-intervention and/or between treatment and control groups post-intervention).

### Study Risk of Bias Assessment

Risk of bias in individual studies was assessed by two of the authors (BW, EG) using the Mixed Method Appraisal Tool (MMAT; Hong et al., 2018). Each study received one point for five items (Table 4); the number of items endorsed indicated the study quality: ‘high’ (4 or 5 of 5 indicators endorsed), ‘moderate’ (3 indicators) or ‘low’ (0–2 indicators).

### Synthesis of Results

Data synthesis and integration first followed the Joanna Briggs Institute Manual for Evidence Synthesis convergent segregated approach (Aromataris & Munn, 2020). This involved initial separate quantitative and qualitative integration, followed by the convergent integrated approach (Aromataris & Munn, 2020; Hong et al., 2018), which “qualitised” quantitative data using thematic analyses. This was conducted using the accepted standard of thematic synthesis, beginning with coding of text line-by-line, then developing descriptive themes and subsequently developing analytical themes which generated new interpretive constructs or explanations; Thomas & Harden, 2008). The qualitised data was then pooled with the qualitative data to produce overarching analytical themes encompassing all studies (Thomas & Harden, 2008). The themes were initially developed by the first author (BW) and discussed, reviewed and agreed upon with the second and last authors (IM, EG). These analytical themes provided an integrated framework for interpreting the mental health/wellbeing outcomes across all studies.

### Certainty Assessment

The Cochrane tool for the Grading of Recommendations Assessment, Development and Evaluation (GRADE; Guyatt et al., 2011) was used to assess risk of bias. A ranking of high, moderate, low or very low certainty based on the pooled evidence is provided in Table 6.

### Community Consultation

The consultation aimed to seek and obtain verbal and written feedback on the findings of the current review and to obtain their perspectives on the extent to which the review’s themes were consistent with their experiences. We also consulted the group with regards to what they thought were meaningful and important recommendations for sibling interventions and supports drawing from and extending the findings of our mixed method review and their lived-in experiences. Consultation was conducted with a Sibling Advisory Panel, with members recruited as part of a larger program of sibling research. Two authors (BW, EG) held a 90-min Zoom semi-structured interview with a group of eight siblings (mean age 16.7 years, age range 9–28 years, four male and four female, two accompanied by their mothers, all Caucasian). Disabled siblings were aged 11 to 26 years, with diagnoses including autism, Down syndrome, cerebral palsy and other neurodevelopmental conditions with genetic origin (i.e. CDKL5 deficiency disorder, Rett syndrome and Trisomy 12p syndrome). These siblings consulted on the language choice in the paper (person or identity first), the ecological validity and relevance of the review’s main findings with regards to their lived-in experiences and were invited to provide their thoughts on recommendations for future sibling interventions and supports and for sibling intervention research.

## Results

### Study Selection

A total of 1893 studies were identified from databases, 108 from registers and 24 from forwards or backwards citation searching (total 2025), of which 1101 were subject to title and abstract screening and 139 to full-text screening (see PRISMA flowchart in Fig. 1). A total of 24 studies were included: 16 quantitative, three mixed methods and five qualitative studies.

### Study Characteristics

Characteristics of participants and interventions are presented in Table 2 (Appendix). Most studies were conducted in the United Kingdom (*k* = 7) and the United States (*k* = 8). Only two lower-income populations were included (Fjermestad et al., 2020 in Cambodia; Phillips, 1999 in the United States for low-income African-American families). Only three studies reported a priori power analyses (Jones et al., 2020; Kang et al., 2021; Zucker et al., 2021) and they were all underpowered to detect moderate effect size differences.

### Siblings in Intervention Groups

Across the 24 studies, there were 915 sibling participants (67% female) in intervention groups with sample sizes ranging from three (McCullough & Simon, 2011) to 99 (Haukeland et al., 2020), with a mean of 37. The sibling ages ranged from 3.5 years (Jones et al., 2020) to 22 years (Calio & Higgins-D’Allessandro, 2021), with a mean of 14.02 years.

### Siblings with NDCs

The siblings with NDCs ranged in age from one to 26 years (where reported, mean 13.34 years; 77% male). Seven studies did not provide characteristics of the disabled sibling other than their NDC. Ten studies focussed exclusively on siblings of autistic children, while NDCs in other studies included ADHD, Down syndrome, cerebral palsy, intellectual disability and combined impairments (i.e. rare neurodevelopmental conditions with or without intellectual and physical disability). Only three studies reported the sibling diagnosis was clinically confirmed (Fjermestad et al., 2019; Lobato & Kao, 2002; Williams et al., 2003), while the remainder were parent report or method was not stated.

### Comparison Groups

None of the qualitative or mixed methods studies included a comparison group. Nine quantitative studies (39%) included a comparison group and seven of these were randomised controlled trials (RCTs). In these nine studies, sibling participants were 4–19 years old (mean 10.5 years; 64% female). Five of the nine studies employed a waitlist-control design; two studies from the same RCT (Jones et al., 2020; Zucker et al., 2021) used an ‘attention-only’ (educational intervention focussing on general adaptive coping skills but not NDCs) participant-blinded controlled intervention; and the remaining two control groups were allocated activities not specific to being a sibling (a free-play outdoor socialisation condition; Kang et al., 2021; other psycho-educational activities; McLinden et al., 1991).

### Intervention Characteristics

Eighteen studies included psychosocial and educational intervention components, with the most common ones being improving knowledge of NDCs and learning adaptive coping skills. Seven studies provided a parallel parent−education program to improve family cohesion and/or knowledge of NDCs. All psychosocial interventions were in group format except one, which ran 1:1 manualised support for school students (Hayden et al., 2019). Two studies ran an intervention in a school setting: after-school group format (Phillips, 1999) or individualised during school (Hayden et al., 2019). Two studies were peer-led support groups (Calio & Higgins-D’Alessandro, 2021; Naylor & Prescott, 2004). One study was run entirely online via the SibChat program (Fell et al., 2022). The most common frequency/duration was one session weekly for 6–8 weeks (*k* = 11), while two studies evaluated a single weekend residential programme and another a 5-day camp (Williams et al., 2003). Most studies employed a pre–post-intervention outcome evaluation design; only seven studies had longer follow-up periods of up to 6 months. Seven studies had a parent−education or participation component (D’Arcy et al., 2005; Fjermestad et al., 2020; Giallo & Gavidia-Payne, 2008; Haukeland et al., 2020; Lobato & Kao, 2002; Roberts et al., 2015; Williams et al., 2003). Fifteen of the 24 studies (62.5% of studies) reported retention rates, ranging from 37 to 89%. Twelve studies (50%) provided reasons for participant attrition, including accessibility, affordability, limited interest and parents being unable to bring their children to the sessions. Only three studies (13%) reported group facilitators’ program fidelity, ranging from 71 to 100%.

### Target Outcomes

Outcomes were thematically condensed into mental health/wellbeing outcomes, including anxiety (*k* = 4), depression (*k* = 6), stress (*k* = 4), self-esteem (*k* = 10), quality of life (*k* = 4), emotional adjustment (*k* = 6), social wellbeing (*k* = 10), family wellbeing (*k* = 8), coping (*k* = 11) and knowledge of NDCs (*k* = 10).

### Outcome Measures

#### *Quantitative *Measures

Across the quantitative studies, 19 different sibling self-report measures were used. Thirteen (72.2%) of the 18 studies used a standardised measure for at least one sibling self-report outcome. The Strengths and Difficulties Questionnaire (SDQ; Goodman, 2001) was the most common measure (*k* = 6; two of these only used the parent−report SDQ), while the Sibling Perception Questionnaire (SPQ; Sahler & Carpenter, 1989), a measure of emotional adjustment, was used in three studies. A measure of self-esteem or self-concept was used in eight studies (see Table 2 for all measures used).

#### Qualitative Measures

Five of the eight qualitative studies used a semi-structured interview with questions focussed on siblings’ knowledge and attitudes towards their sibling’s NDC, exploring feelings, experiences and challenges common to being a sibling of an individual with a NDC (Calio & Higgins-D’Allessandro, 2021; D’Arcy et al., 2005; Hayden et al., 2019). Two studies evaluated wellbeing outcomes via informal dialogue with siblings in group session format focussing on self-worth and inner value (McCullough & Simon, 2011; Naylor & Prescott, 2004). Fjermestad et al. (2019) used both a clinician-rated behavioural observation coding system and group recorded discussions.

### Individual Studies’ Findings on Pre–Post-Intervention Changes in Sibling Target Outcomes

Individual study results are presented in Table 3.

Seventeen of the 24 included studies (70.83%) reported post-intervention improvements in at least one outcome. Five out of the 24 studies (20.83%) did not find any statistically significant post-intervention improvement in any outcomes, although some non-statistically significant improvements on coping skills, quality of life or social wellbeing were noted (D’Arcy et al., 2005; Fjermestad et al., 2020; McLinden et al., 1991; Naylor & Prescott, 2004; Smith & Perry, 2005). Two quantitative studies reported negative effects on at least one sibling outcome: Gettings et al. (2015) reported worse quality of life pre–post-intervention for five out of six siblings in the intervention group, while Jones et al. (2020) reported increased anxiety in both intervention and comparison groups pre–post-intervention.

### Group Comparison Post-intervention Findings

Five out of the nine studies with comparison groups reported the intervention group made larger improvements compared to the comparison or waitlist groups which showed minimal changes. The other four studies using a non-waitlist comparison had mixed results: the intervention group improved non-significantly (Jones et al., 2020) and significantly (Kang et al., 2021; Zucker et al., 2021) compared to the control group post-intervention; McLinden et al. (1991) found the intervention group improved more than the comparison group on social support and positive affect, while the comparison group improved on self-esteem. Overall, therefore, all nine studies reported small to large benefits in favour of the intervention group in at least one outcome measure.

### Long-Term Follow-Up Outcome Findings

Of the four studies measuring post-intervention outcomes for more than three months, one reported significant sustained improvements for the intervention group up to 12 months’ post-intervention on self-esteem, mood, negative adjustment and behaviour problems, while the comparison groups did not maintain significant improvements at 9 or 12 months (Williams et al., 2003). Two other studies reported that the intervention group did not maintain improvement at 3 months for self-esteem or coping skills (Lobato & Kao, 2002; Roberts et al., 2015) and scores regressed to baseline pre-intervention in both studies. Finally, one study without a comparison found significantly improved sibling emotional adjustment, communication and knowledge of disability which was maintained at 4- and 7-months follow-up (Haukeland et al., 2020).

### Summary of Individual Study Findings

Overall, the most consistently positive sibling self-report outcomes in this review derived from the five studies which included a comparison group and follow-up data. These studies found larger positive effects on self-esteem, family wellbeing and knowledge of disability post-intervention and consistently indicated that siblings in the intervention groups appeared to benefit more than those in the comparison group (Giallo & Gavidia-Payne, 2008; Phillips, 1999; Williams et al., 2003). Siblings in interventions with combined psychosocial and educational components appeared to have better outcomes post-intervention than those with only social activities (Brouzos et al., 2017; Haukeland et al., 2020; Williams et al., 2003; Zucker et al., 2021), as were siblings in programs with parallel parenting education (compared to studies without parental involvement; Lobato & Kao, 2002; Roberts et al., 2015).

### Risk of Bias of Individual Studies

The MMAT quality assessment (see Table 4) indicated that most included intervention studies were moderate or low quality (i.e. high risk of bias), with quality scores ranging from 20 to 80%. Of the qualitative studies, Fjermestad et al. (2019) and Calio and Higgins-D’Allessandro (2021) were rated as high quality (80%). Four of the five quantitative RCTs were rated moderate quality (60%; medium level of bias). Only seven of the 24 studies described the group facilitators and their qualifications in detail, hence an assessment of clinician bias was not possible. Generalisability was considered low across studies, as most siblings were Caucasian, from high income countries and with parents who were engaged with the intervention.

### Synthesis of Results from All Included Intervention Studies

The synthesis of qualitative and quantitative data resulted in four analytical themes encompassing the following outcomes: emotional and behavioural adjustment (*k* = 14, including anxiety, depression and emotional adjustment), knowledge and education (*k* = 14, including NDC knowledge and coping), inter-personal wellbeing (*k* = 14, including family and social wellbeing) and intra-personal wellbeing (*k* = 19, including self-esteem, stress and quality of life) (Table 5).

Our review synthesis showed that the strongest positive improvements for siblings across all included intervention studies were noted for increasing knowledge of NDCs (*k* = 10) and improving self-esteem post-intervention (*k* = 8). The sibling wellbeing domains that appeared to show fewer improvements post-interventions were anxiety (*k* = 2 improved, 2 little/no change, 1 decline), depression (*k* = 3 improved, 2 no change) and quality of life (*k* = 2 improved, 1 no change, 1 decline).

#### Theme 1: Emotional and Behavioural Adjustment

Adjustment was measured with seven different instruments in ten quantitative studies and explored in four qualitative studies. Seven studies using the self-report SDQ and SPQ reported statistically significant or non-significant improvements post-intervention, except for one study which found non-significant worsening of emotional adjustment (Gettings et al., 2015). Four studies found significant small reductions in the non-disabled siblings’ self-reported problem behaviours post-intervention (Hayden et al., 2019; Lobato & Kao, 2002; Roberts et al., 2015; Williams et al., 2003), while two studies did not find any significant changes post-intervention (McLinden et al., 1991; Smith & Perry, 2005).

All four qualitative studies reported improvements in emotional adjustment, including learning emotion regulation techniques (Fjermestad et al., 2019), improving mood (D’Arcy et al., 2005), reducing negative attitudes towards their sibling (Naylor & Prescott, 2004) and reducing anxiety (Calio & Higgins-D’Allessandro, 2021).

#### Theme 2: Knowledge and Education

All 13 studies measuring knowledge of NDCs reported improvements. Seven studies used a parallel parent education component to improve parent-reported understanding of siblings’ needs and parent–child communication. In two intervention groups, learning about autism and building coping strategies led to significant improvements in sibling-reported sibling relationship quality (Zucker et al., 2021) and psychological functioning (Jones et al., 2020), not evidenced in the active comparison groups without an autism-focussed psychoeducational component. Coping skills were also targeted using psychoeducation; these were measured in five quantitative, two mixed methods and all qualitative studies. Quantitative studies reported reductions in maladaptive coping (Giallo & Gavidia-Payne, 2008; Roberts et al., 2015) and/or increases in adaptive coping (Hayden et al., 2019; Jones et al., 2020). The five qualitative studies all found improvements in sibling-reported coping strategies.

#### Theme 3: Inter-personal Wellbeing

Inter-personal wellbeing comprises peer, sibling dyad, parent–child and family-wide communication and cohesion. Eight quantitative studies and four qualitative studies measured family functioning. Of the quantitative studies, six reported significant small improvements in family functioning and only two reported no improvement post-intervention (Fjermestad et al., 2020; Philips, 1999). The quality of the sibling−dyad relationship improved in three of the six studies measuring this outcome (Evans et al., 2001; McLinden et al., 1991; Zucker et al., 2021). Negative attitudes of the non-NDC sibling towards their sibling decreased in three studies (Haukeland et al., 2020; Lobato & Kao, 2002; Williams et al., 2003). Seven of the eight quantitative studies found improvements in positive peer and/or teacher relationships. All four qualitative studies also found siblings reported improvements in family wellbeing and peer relationships. Changes in social support were measured in six quantitative studies and one mixed methods study; five found post-intervention increases in the siblings’ perceived support; one study did not find any change post-intervention or between groups (Giallo & Gavidia-Payne, 2008).

#### Theme 4: Intra-personal Wellbeing

The seven quantitative studies and one mixed method study measuring self-esteem or self-concept used six different measures, with five studies finding a significant improvement post-intervention (Evans et al., 2001; Kang et al., 2021; Phillips, 1999; Smith & Perry, 2005; Williams et al., 2003) and two studies finding no significant changes post-treatment and no group differences (D’Arcy et al., 2005; McLinden et al., 1991). One study measuring quality of life found a significant decline (i.e. poorer quality of life) post-intervention (Gettings et al., 2015). Themes identified post-intervention in the three qualitative studies exploring intra-personal wellbeing indicated improvements in self-esteem, self-worth, self-concept, autonomy and feelings of bravery and resilience (Calio & Higgins-D’Allessandro, 2021; McCullough & Simon, 2011; Rye et al., 2018).

### Comparison of Parent-Reported and Sibling Self-report Outcomes

To contextualise the self-report outcome findings presented in this review, we briefly explore in this section how these compare to the more commonly studied and reported parent-reported outcomes in the studies which included these. Ten studies included one or more parent−report measures of sibling emotional and behavioural functioning, with parents consistently reporting larger sibling improvements in mental health post-intervention as compared to sibling self-report. Five of the ten studies used the parent-reported SDQ, all finding improvements post-intervention and significant group differences favouring the intervention group (Fjermestad et al., 2020; Giallo & Gavidia-Payne, 2008; Haukeland et al., 2020; Hayden et al., 2019; Roberts et al., 2015). Of these five studies, three also measured sibling self-report SDQ and contrastingly to the parents, the siblings either did not report significant improvements (Fjermestad et al., 2020; Roberts et al., 2015) or reported similar improvements to paternal but not maternal report in one study (Haukeland et al., 2020).

### Sibling Experiences and Perceptions of the Interventions/Support Programmes Received

Eighteen of the 24 (75%) studies examined the siblings’ overall satisfaction with the intervention. All 18 evaluations were positive, with siblings reporting enjoyment, improved knowledge of NDCs, meaningfulness or personal development. In six out of the 24 included studies, a minority of siblings also provided some negative feedback/evaluation points, in which other participant’ behaviours (interrupting, pressuring to speak; Fjermestad et al., 2020) were noted and one sibling suggested the need for greater ethnic diversity (Fell et al., 2022).

### Pre-intervention Risk and Resilience-Promoting Factors Associated with Post-intervention Outcomes

Siblings with lower baseline self-reported resilience-promoting factors (such as fewer coping skills and lower self-esteem) tended to show the most improvements post-intervention in the 11 quantitative and three qualitative studies exploring these (Jones et al., 2020; Smith & Perry, 2005). Three studies with siblings with baseline parent-reported ratings on emotional and behavioural adjustment in the normative range also found significant, yet smaller, pre–post-intervention improvements. The largest positive intervention results tended to include siblings of a younger age (mean age of less than 11 years) and samples with more male than female participants (Brouzos et al., 2017; Kryzak et al., 2014). However, these patterns were contradicted in some studies: a study with all females reported positive changes in psychosocial adjustment (Rye et al., 2018); a study of siblings aged 20–22 reported significant improvements in wellbeing (Calio & Higgins-D’Allessandro, 2021), while adolescents (aged above 12) improved more on self-esteem than children (Williams et al., 2003).

Ethnicity and socioeconomic status were not explored, as studies reporting these variables (*k* = 11) mostly included Caucasian, higher socioeconomic status participants.

Regarding the type of NDC, the studies with larger positive effects tended to include siblings of individuals from a heterogeneous group of NDCs compared to studies with siblings of individuals with one NDC such as autism (Giallo & Gavidia-Payne, 2008; Haukeland et al., 2020; Phillips, 1999; Williams et al., 2003). However, this difference may be due to the severity of the NDC or behavioural problems of the child with the NDC, rather than syndrome/condition specificity (Jones et al., 2020). Additionally, these studies have different intervention components and measures, which may result in a trend towards more positive results.

### Certainty of Evidence

Confidence in the body of evidence per outcome is presented in Table 6. The most certainty in the overall evidence was rated high for self-esteem, social wellbeing, family wellbeing and NDC knowledge. The quality of life outcome was rated very low due to small sample sizes, non-standardised or heterogeneous instruments used and inconsistent results.

### Sibling Community Consultation Findings

The Sibling Advisory Panel were presented with the key review findings in a lay presentation and were invited to share their thoughts and feedback. Sibling community participants agreed with the presented outcomes. Most siblings had previously participated in a family intervention targeting their disabled sibling, which they had found beneficial for improving family communication.

There were two main recommendations. First, siblings shared that at school they experienced bullying, segregation, social isolation, stigma and the staff were under-skilled to support siblings’ unique needs or educate other students on disabilities. One sibling stated intervention targets in schools could include “compassion and kindness, improved self-esteem, improved social relationships with friends, less feelings of shame and greater support within the school environment”. Therefore, a school-based psycho-educational intervention for peers and staff may be beneficial to foster acceptance and understanding. A second recommendation was regarding the burden of responsibility and increasing anxiety with age; siblings indicated there should be peer-led support groups and education on future planning offered to older siblings.

## Discussion

Overall, the current review extends existing evidence on psychosocial interventions for siblings in three ways: firstly, by combining quantitative with qualitative studies and a community consultation to evaluate siblings’ experiences; secondly, by differentiating parent and sibling reported outcomes and focussing on and highlighting sibling self-reported outcomes; and thirdly by examining a broader age range of siblings of individuals with NDCs, as compared to previous reviews which focussed on younger siblings of individuals with mixed physical/medical/psychiatric conditions and NDCs. As this review identified 24 studies meeting inclusion criteria, the results should be interpreted with caution and our discussion provides a synthesis of these findings on the premise that further research is required to draw stronger conclusions regarding sibling outcomes and intervention effectiveness.

### Siblings’ Self-reported Mental Health Outcomes Following Psychosocial Interventions

The current review of 24 intervention studies identified ten sibling mental health/wellbeing outcomes studied in intervention research to date: anxiety, depression, stress, self-esteem, quality of life, emotional adjustment, social wellbeing, family wellbeing, coping and NDC knowledge. Four analytical themes encompassing these outcomes derived from both quantitative and qualitative results and the sibling community involvement included (i) emotional and behavioural adjustment, (ii) education and knowledge, (iii) inter-personal wellbeing and (iv) intra-personal wellbeing.

The largest immediate post-intervention improvements were in self-esteem, social wellbeing and knowledge of NDCs. The most sustained improvements at follow-up periods in the few studies that explored these were in emotional and behavioural adjustment and in NDC knowledge. The outcomes with less consistent findings and smaller post-intervention or group differences were anxiety, depression and quality of life, although this may also have been due to floor effects or selection bias amongst sibling participants. The intervention groups generally had better outcomes than the waitlist control and active comparison groups.

### Siblings’ Qualitative Experiences and Perceptions of the Interventions

In general, qualitative studies reported more positive intervention effects than quantitative studies on sibling wellbeing outcomes (Rye et al., 2018) It may be that standardised measures used in current intervention studies are not capturing outcomes considered important to siblings (Calio & Higgins-D’Allessandro, 2021). It is also possible that siblings may be providing socially desirable answers in the interviews, although three studies had anonymous feedback forms and in interviews asking for both positives and negatives of the intervention, the positives outweighed the negatives (Evans et al., 2001; Fjermestad et al., 2020; Giallo & Gavidia-Payne, 2008).

Qualitative results from the sibling stakeholder consultation resulted in two additional recommendations: (i) interventions within the school context are needed to improve acceptance, compassion and understanding and (ii) education or support programs exploring and supporting future planning responsibilities targeting older siblings to reduce anxiety and worries about the future. Overall, siblings reported benefitting from interventions and it may be that mixed methods of data collection or different instruments are needed to capture the spectrum of sibling experiences.

### Pre-intervention Risk and Resilience-Promoting Factors Associated with Intervention Outcomes

This research question remained largely unanswered due to non-reporting or large heterogeneity of demographic information/sample characteristics and few studies reporting pre-intervention cognitive, emotional or behavioural functioning. Trends in included studies indicated that sibling participants of younger ages (D’Arcy et al., 2005; Hayden et al., 2019; Zucker et al., 2021), male siblings (Brouzos et al., 2017; Lobato & Kao, 2002), siblings of individuals with lower disability symptom severity (Jones et al., 2020) and siblings with poorer baseline (clinically elevated) functioning as reported by parents (Giallo & Gavidia-Payne, 2008; Roberts et al., 2015), tended to report more improvements from the interventions or support groups. However, poorer baseline functioning may have caused ‘ceiling effects’ (Giallo & Gavidia-Payne, 2008; McLinden et al., 1991). No quantitative studies adopted a strengths-based approach by measuring positive impacts of having a sibling with a disability or baseline individual-level resilience-promoting factors (e.g. trait resilience, optimism, emotion regulation) which may be predictors of benefit from intervention (Green, 2013). This is problematic, as support groups may be targeting siblings who may not need or benefit from targeted interventions and may be missing key risk groups of siblings (Tudor & Lerner, 2015).

### The Relationship Between Intervention Approaches and Sibling Outcomes

Interventions in group format held weekly for 6–8 weeks, with 60–90 min sessions, appeared to have more positive outcomes (Brouzos et al., 2017; Kryzak et al., 2014; Lobato & Kao, 2002; Roberts et al., 2015). However, there was minimal consensus in the approach adopted in interventions, even for those using similar manualised programs, although it is apparent that group designs were most widely used (21 of 24 studies) and produced some positive outcomes for siblings (D’Arcy et al., 2005; Kryzak et al., 2014; Lobato & Kao, 2002; Roberts et al., 2015). Due to this heterogeneity, it is difficult to compare the influences of different types or approaches to interventions on sibling outcomes (McKenzie Smith et al., 2018). As also commented in previous reviews, no psychosocial intervention included in this review qualifies as ‘well-established’ or ‘probably efficacious’ (Tudor & Lerner, 2015) and none of the intervention studies included were a ‘high quality’ randomised controlled trial (Higgins & Green, 2011). However, a key intervention component common in several of the studies reporting some improvements/positive outcomes was parental involvement. Seven studies used a parallel parent education component with all but one (Fjermestad et al., 2020) reporting significant improvements in outcomes including NDC knowledge, self-esteem, family communication, positive attitudes and parent-reported behaviour problems (D’Arcy et al., 2005; Giallo & Gavidia-Payne, 2008; Haukeland et al., 2020; Lobato & Kao, 2002; Roberts et al., 2015; Williams et al., 2003). In two of these seven studies which included parent reflections on the interventions (Giallo & Gavidia-Payne, 2008; Roberts et al., 2015), parents shared that they were often not aware of the negative impact of the NDC on the non-disabled sibling and described improved parenting skills and family communication post-intervention (Fjermestad et al., 2019; Gettings et al., 2015; Giallo & Gavidia-Payne, 2008; Haukeland et al., 2020; Lobato & Kao, 2002). Collectively, this evidence suggests the involvement of parents in sibling intervention programs and parallel parent education programs, may be an important ingredient. The discrepancy between parent and sibling reported outcomes was evident in seven of the included studies that assessed both, with parents generally reporting larger sibling improvements in mental health post-intervention compared to sibling self-report (Fjermestad et al., 2020; Giallo & Gavidia-Payne, 2008; Haukeland et al., 2020; Hayden et al., 2019; Roberts et al., 2015). These highlights the crucial role of self-reported experiences being central in future intervention studies’ primary outcomes and questions the sensitivity of parents as sole informants (Lobato & Kao, 2002).

### Comparison of the Present Review’s Findings with Previous Reviews

Our review included 13 studies not included in the five existing reviews (Hartling et al., 2014; Kirchhofer et al., 2022; McKenzie Smith et al., 2018; Thomas et al., 2016; Tudor & Lerner, 2015). Similar to the previous systematic reviews, our study found overall positive effects of interventions in over 65% of the included studies, primarily for self-esteem, social support and knowledge of NDCs. In contrast to all five earlier reviews, however, the current review found little evidence for sibling self-reported improvements in anxiety, depression or quality of life. The finding in this review that parents consistently reported greater sibling emotional and behavioural improvements following interventions as compared to siblings’ self-reports is difficult to interpret, as all earlier reviews combined parent and sibling reports and the heterogeneity across studies makes comparisons difficult. Our focus on sibling self-report; therefore, revealed unique sibling needs and experiences not explored in previous studies or reviews. In addition, the qualitative components of studies included in this review, in addition to our community consultation, identified new sub-themes from the data: specifically, we found evidence for improved intra-personal wellbeing including enhanced resilience and self-worth (Calio & Higgins-D’Allessandro, 2021; Rye et al., 2018) not evident in previous reviews evaluating quantitative outcomes.

### Methodological Limitations of Included Studies

The primary limitation across studies was under- or non-reporting of key variables; few studies reported family demographics and many included heterogeneous participant characteristics. The use of non-standardised or heterogeneous instruments may have contributed to contradictory results, such as reduced quality of life found in Gettings et al. (2015). Most studies only reported mean scores rather than using measures which could report the proportion of siblings in borderline or clinically elevated range pre- and post-intervention, which would have provided more insight into baseline characteristics of siblings benefitting most from interventions (Giallo & Gavidia-Payne, 2008; Ma et al., 2017). All included studies only assessed one sibling in the family, commonly the sibling closest in age to the target child, which may introduce sampling bias (Ma et al., 2017).

As many studies used a within-subjects pre–post design with small samples and without a comparison group (Kryzak et al., 2014; Lobato & Kao, 2002; Smith & Perry, 2005), no assumptions about superiority of sibling interventions over other forms of support or services which may also have positive effects can be made (Fjermestad et al., 2020). Additionally, using comparison groups of siblings receiving individualised or specific components of interventions would enable mechanisms of change and intervention components to be identified (Roberts et al., 2015). The small sample sizes and non-reporting of data did not allow analysis of sub-groups to identify any moderator effects in this review. There are likely groups of higher-risk siblings experiencing a range of individual, family or broader sociocultural/structural risk factors, who may benefit more from personalised/individual interventions and a broader range of supports and services.

Replicability of the intervention design would be problematic, as most studies did not report key components of the interventions or interview protocols. There was a lack of empirical evidence behind intervention design, other than the use of psychoeducation. In the current review, most studies did not include a follow-up and those which did had high attrition (Haukeland et al., 2020). Clinical significance, not simply statistical significance, should be assessed in more studies; only one study evaluated clinically meaningful change in individual siblings (Giallo & Gavidia-Payne, 2008).

### Limitations of the Current Review

The current review has limited generalisability due to heterogeneity of reported family demographics, small sample sizes and non-reporting of data which did not allow this review to conduct meta-analyses or quantitative moderation analyses of sub-groups. There are likely groups of higher-risk siblings who may benefit more from psychosocial interventions (McKenzie Smith et al., 2018; Tudor & Lerner, 2015). For instance, the type and severity of NDC of the disabled child likely has differential impacts on siblings (Mandleco & Webb, 2015). In the current review, included studies emphasised that having an autistic sibling may present unique challenges distinct from other NDCs (Zucker et al., 2021), consistent with prior literature (Kaminsky & Dewey, 2002; Orsmond & Seltzer, 2007). In the sibling community consultation, all participant siblings were Caucasian, middle to upper socioeconomic status and with educated parents; most were older than their diagnosed sibling and most siblings with NDCs were autistic, hence the sibling community consultation group is not representative of all siblings of people with NDCs.

### Future Directions

Methodologically, the primary future direction for future intervention/support studies is greater replicability and stronger evidence drawn from larger sample sizes for sibling reported outcomes. Replication studies evaluating the same type of intervention, with the same study population and outcome measures with repeated assessments are needed to enable comparisons across studies (Tudor & Lerner, 2015). The use of active comparison and waitlist control groups with a larger sample size in all intervention studies will further improve the clinical utility of controlled trials (Hartling et al., 2014). Studies should further explore and report other possible moderating variables or demographics known to impact sibling psychological functioning and appropriate power analyses should be conducted to allow testing of such research questions. There is also a need for a more collaborative approach across research teams and community, clinical or school-based supports.

Secondly, in addition to tailoring interventions based on sibling/family context and accessibility, the evidence from this review and the sibling community consultation suggests tailored interventions may be beneficial for siblings at different life periods. For instance, younger children tended to show greater improvements on knowledge of NDC (Brouzos et al., 2017), whereas adolescents improved more on self-perception and self-esteem (Williams et al., 2003). The oldest participant across identified studies was aged 22, indicating a lack of intervention studies for older siblings in emerging adulthood. Older siblings have increased anxiety regarding caregiving responsibilities as their disabled sibling ages (Macks & Reeve, 2007; Stampoltzis et al., 2014) and they benefit from support programs targeting sibling empowerment and community connection (Burke et al., 2020). Other stages are important to consider, such as immediately after the initial diagnosis of the disabled sibling and at key developmental transition periods (Hastings, 2016; Petalas et al., 2009). Other family characteristics or dynamics (such as family cohesion, family stressors, family structure, size and resources; see section “Introduction”) may be moderating variables impacting mental health outcomes and although these were not explored or targeted in the included studies, it is important that future multi-targeted support programmes are developed and systematically evaluated, over and above programmes targeting solely the siblings.

Finally, accessibility of interventions across siblings from all socioeconomic and ethnic backgrounds should be considered. Further, our results indicated that in six studies, siblings were unable to access services due to parental time or financial constraints (Giallo & Gavidia-Payne, 2008); previous reviews also emphasise problems with reliance on clinic-based services with waiting lists, family financial strain or fear of causing additional stress for parents (Green, 2013). This review found that only two studies targeted non-Caucasian ethnicities or low-income families and these studies suggested that these families may have unique negative experiences due to disadvantage, stigma or cultural expectations (Fjermestad et al., 2019; Phillips, 1999). As only one study occurred in a school context (Hayden et al., 2019), further research is required into programs delivered in education settings and via more accessible service providers.

#### Proposed Novel Targets for Sibling Interventions Based on Review Outcomes

Two further novel future directions for sibling interventions are made based on the results of this review which indicated that knowledge, coping and self-esteem were most amenable to change. Firstly, improving emotion regulation skills as a form of coping may be a worthwhile target to consider in future interventions. Knowledge and coping are inter-related and both linked to improved psychosocial wellbeing (Lobato & Kao, 2002). Emotion dysregulation is a pervasive transdiagnostic risk factor for maladaptive outcomes, which can be improved through enhancing helpful coping skills (Fjermestad et al., 2019). Although not all siblings experience difficulties in these domains, they are risk factors for adjustment difficulties and could be explored further in future support programmes.

Second, it was evident from the qualitative studies that many siblings were often highly self-critical, anxious and struggled with burden of perceived responsibility, while quantitative results indicated they had poor baseline global self-worth (McCullough & Simon, 2011; Naylor & Prescott, 2004). Observational sibling studies have found that self-criticism contributes to greater risk for internalising difficulties (Hwang & Charnley, 2010; Murrin et al., 2020). Psychological interventions, such as self-compassion therapy have been directly related to decreased self-criticism, improved self-esteem and less treatment drop-out amongst siblings (Kılıç et al., 2020) and such efforts could be more systematically evaluated in future intervention studies.

## Conclusion

The present review is the first to focus on and comprehensively examine self-reported sibling mental health outcomes following interventions or support programmes targeting their wellbeing. We found that although these interventions do generally improve sibling wellbeing domains, there remain significant limitations in evaluating these outcomes. We suggest future directions focus on overcoming methodological limitations and improving replicability, tailoring interventions based on sibling and family context and improving accessibility of interventions for all siblings; we further provided suggested novel targets for individual-level sibling psychosocial interventions targeting emotion regulation and self-compassion. Which siblings require support and what type of support they may most benefit from, should be assessed on an individualised basis; more data are needed to inform these clinical decisions. Future research could explore whether sibling psychosocial interventions should be delivered as treatment (for siblings with clinically elevated baseline difficulties), as indicated prevention (for siblings with pre-intervention borderline difficulties) or as universal prevention (for any/all siblings; Haukeland et al., 2020). Overall, psychosocial interventions reviewed in this study did produce some improvements in some outcomes for siblings, however continuing targeted intervention/support evaluation research will allow continuing enhancement of sibling psychological assets whilst supporting mental health and wellbeing of those most in need.

## Additional Information

This systematic review was pre-registered on Prospero, CRD42021264744. The PRISMA-P checklist was followed when preparing the protocol.

## Data Availability

All data extraction Excel spreadsheets, search chains, tables included in the appendices and a list of studies narrowly missing the inclusion criteria are publicly available on the first author’s OSF page, accessible online: osf.io/u4gaj.

## Appendix

See Tables 1, 2, 3, 4, 5, 6.

**Table 1:** Search Terms used for all databases

1. Outcome terms (combine with 6, below)
Title: (mental health) or wellbeing or well-being or (well being) or (quality of life) or (psychiatric disord*) or (psychiatric prob*) or (mental ill*) or (mental disord*) or psychopatholog* or (emotional prob*) or (emotional disord*) or (behavio#ral prob*) or (behavio#ral disord*) or (psychological prob*) or DSM* or ICD* or (psychiatric* disord*) or psychosis or psychotic or anxi* or depress* or oppositional or hyperactiv* or conduct disord* or obsess* or phobi* or mood or schizophren* or bipolar or anorex* or bulimi* or (eating disorder) or (challenging beh*) or (internali#ing or externali#ing) or (mental health diagnos#s) or (affective disorder) or (personality disorder) or adjustment or coping or adaptation or psychosocial or (health related quality of life) or (quality of life) or (quality adjusted life year) or “QALY”
2. Developmental condition terms
Title: (*development* disorder) or (*development* condition) or (*development* delay) or syndrome or disabilit* or autis* or ASD or Asperger* or (Pervasive Developmental Disorder) or impairment or (Cerebral Palsy) or (rare disorder) or (mental retard*) or (mentally retard*) or (mental* handicap*) or neurogenetic or (chronic illness) or (chronic condition)
Repeat search adding the following: or Angelman or CDKL5 or congenital or (Fragile X) or (Prader-Willi) or (Prader-Labhart-Willi) or Rett or (Tuberous Sclerosis) or (22q11*) or (Williams syndrome) or (Williams-Beuren syndrome) or (Down syndrome) or (trisomy 21) or (Down#s syndrome) or (learning disabilit*) or (learning disord*) or (intellectual disabilit*) or (f#etal alcohol spectrum disorder) or (FASD) or (velo‐cardio‐facial syndrome) or Angelman or (tuberous sclerosis complex) or (Cornelia de Lange) or (22q13*) or (Phelan#McDermid) or SHANK3 or (velocardiofacial) or (DiGeorge syndrome) or (deletion syndrome) or (regressive disorder) or (neuropsychiatric decompensation)
3. Sibling terms
Title: Sibling* or brother* or sister* or kin or famil*
Abstract: Sibling* or brother* or sister*
4. Intervention terms (combine with 1, 2, 3 above)
Abstract: intervention or (early intervention) or support or (self-help) or psychotherapy or psycho-education or group* or (group intervention) or (school based intervention) or (social support) or treatment or program* or (mental health service*)

Searches were run using combinations of concept terms, e.g. “1 and 2 and 3 and 4”

Note that hashtag # is used in place of * in some databases. MeSH terms were added in each database

The search strategy was refined several times without altering the initial search strategy (e.g. the terminology for referring to disabled people has changed over time, adding the word ‘handicap*’ yielded additional hits but not alter the results). If an alteration for one database did not yield additional hits, it was not adjusted for other databases. This process of refining the search strategy is an accepted practice in disability research (see Kruithof et al., 2020)

**Table 2:** Characteristics of participants and interventions in the included studies

Study (year) Country	Sibling with an NDC *N* Mean age (range) Male/female NDC type	Intervention group siblings *N* Mean age (range) Male/female Other data	Comparison group siblings *N* Mean age (range) Male/female Other data	Intervention: Duration, design, components, and assessment times	Intervention target outcomes as stated in each study’s aims	Measures (informant, construct measured)
Quantitative studies						
Brouzos et al. (2017) Greece	38 N/R 77.3%M All autistic	12 10.75 (6–15) 10 M, 2F 50% older than disabled sibling	16 10.75 (6–15) 8 M, 8F 56.3% older Waitlist control	8 × 90 min sessions Psycho-education, emotional education and regulation, cognitive restructuring, problem-solving Pre/post-intervention only	Coping skills Knowledge of autism	SDQ (sibling; emotional adjustment) Coping/Adjustment scale Knowledge of autism questionnaire No intervention evaluation survey
Evans et al. (2001) U.K	No details provided other than had ‘learning disabilities’ with or without challenging behaviours	2 groups 1st group: 9 8–12 years 6F, 3 M 2nd group: 9 7–12 years 7F, 2 M	None	3 consecutive full days during school holidays, then weekly for 6 evenings, and 1 final full day on weekend. Structured program with age-appropriate activities Pre/post-intervention only	Problem-solving Coping skills Creativity	Culture Free Self-Esteem Test Knowledge of NDC questionnaire Evaluation survey CBCL (parent, baseline only; emotional adjustment)
Fjermestad et al. (2020) Cambodia	54 9.6 (3–16) 56% M DS, ADHD, autism, ID, LD	52 12.7 (8–21) 44% F SES rated good (11.9%), medium (54.8%), poor (33.3%)	None	Manualised, 1 full day of 5 sessions (1 × 20 min, 4 × 60 min): 3 sessions parallel group sessions for siblings and parents. 2 sessions integrated sibling-parent dialogues 4-month follow-up	Emotional and cognitive-behavioural regulation Parent–child communication	SDQ (sibling and parent) DASS (parent self-report; depression) PCCS (child and parent; communication) Intervention evaluation survey (post-intervention and 4 month follow-up)
Gettings et al. (2015) U.K	6 11–13 years N/S All autistic	6 8–13 years 5F, 1 M	None	4 face to face, 4 audio-conference, weekly 1 h for 8 weeks. Topics on group cohesion, problem-solving, psychoeducation, hope One follow-up interviews at 4–6 months	Understanding of NDC Forming new connections Emotion regulation	SDQ (sibling and parent) PedsQL4.0 (quality of life) Sibling’s Views Questionnaire (knowledge) Intervention evaluation survey
Giallo and Gavidia-Payne (2008) Australia	Disabled siblings of intervention children: 12 10.92 (3–16) 58.2%M NDC n(%): DS 1 (8.3), Autism 3 (25.0), ADHD 1 (8.3), Polymicrogyria 1 (8.3), Multiple 2 (16.7), Congenital Heart Disorder 2 (16.7), WS 1 (8.3) NDC severity (parent-rated): Mild 3 (25.0), moderate 8 (66.7), severe 1 (8.3)	12 11.75 (9–16) 50%M 58.3% older than disabled sibling	9 11 (8–16) 33.3%M 88.9% older Waitlist control NDC siblings of control group children: *n* = 9 10.07 (6–21), 55.6% M NDC *n* (%): DS 3 (33.3), Autism 2 (22.2), Multiple 2 (22.2), CF 1 (11.1), WS 1 (11.1)	RCT SibStars program with waitlist control. Psycho-educational components, 6 × weekly 20–30 min sessions, with telephone support. Parallel parent−education program Pre/post-intervention only	Problem-solving, stress reduction, manage emotional reactions	Sibling Daily Hassles and Uplift Scale (stress) Self-Report Coping Scale SDQ (parent) Participant Satisfaction Questionnaire (custom intervention evaluation survey)
Haukeland et al. (2020) Norway	99 10.4 (3–21) 45%F Caucasian NDC (%) rare disorder with ID (29.3), Autism (25.3), rare disorder with physical impairment (23.2), Congenital heart disease (12.1), DS (7.1), CP (3.0)	99 11.5 (8–16) 54.5% F 59.8% older than disabled sibling	None	Joint sibling-parent manual-based program, 5 sessions within 2–5 day samples. 22 groups with 3–7 siblings per group Parallel parent−education program T2 (*n* = 77) 18 weeks after completion. T3 (*n* = 55) 33 weeks after completion.	Improve knowledge of NDC, parent–child communication, emotion expression and regulation	SDQ (parent and sibling) PCCS (sibling) SPQ (sibling; negative adjustment sub-scale, NAS) Acceptability and Satisfaction Evaluation form (custom intervention evaluation survey)
Jones et al. (2020) U.S	44 7.43(3.37) (3–17) All autistic Support group: 3F, 18 M Control group: 5F, 18 M	24 8.31(3.52) (3.5–18) 10F, 14 M 40% older than disabled sibling 4 white, 3 mixed race, 13 Asian, 2 Hispanic, 1 Guyanese	30 8.62(3.75) (4–15) 20F, 10 M (15 older, 13 younger, 2 same age) 6 white, 6 mixed race, 10 Asian, 6 Hispanic, 1 Guyanese Attention-only condition	RCT of pilot program (Kryzak, 2014) Support group: 2 h per week, 10 weeks, education in social, communication and problem-solving skills Attention condition did not include autism education Autistic siblings all received parallel intervention	Problem-solving, communication, emotion regulation	CDI-2 (depression) RCMAS-2 (anxiety) CCSC (coping) No intervention evaluation survey
Kang et al. (2021) South Korea	All had ‘developmental disabilities’ no further details reported	18 9.17 (7–13) 11 M, 7F	11 10.27 (7–13) 7 M, 4F Control ‘free play’ condition (also waitlist)	RCT. Weekly art therapy in the forest for 8 weeks, 60 min per session. Waitlist control group with a free-play outside condition Pre/post-intervention only	Physiological and emotional conflict resolution, forming connection, self-esteem, stress reduction	Stress sale Self-esteem scale Attention quotient and anti-stress quotient (EEG measures) No intervention evaluation survey
Kryzak et al. (2014) U.S	1st group: *n* = 7 5–11 years 4 M, 3F 2nd group: *n* = 8 4–13 years 8 M All autistic	1st group: *n* = 6 6–18 years 4 M, 2F 2nd group: *n* = 9 5 M, 4F 6–14 years	None	‘Support and Skills Program’ (SSP), 2 h on Saturdays over 8 weeks (1st group) and 9 weeks (2nd group). Individualised skills session plus recreation time. Weekly topic focussed on a characteristic of autism and coping skills Parallel individualised skills interventions for the autistic child	Coping skills, forming connection, emotion education Behavioural observations of positive and negative affect (frequency, time) coded from videos of sessions	CDI (depression) RCMAS-2 (anxiety) Autism Sibling Knowledge questionnaire Social validity questionnaire No intervention evaluation survey
Lobato and Kao (2002) U.S	44 8.7 (1–16 years) 33 M, 14F NDCs: physical disabilities (26%), autism (23%), MR (21%), medical coditions (17%), combined psychiatric and LD (13%)	54 9.8 (8–13) 24 M, 30F 57% older than disabled sibling 89% Caucasian	None	Manualised SibLink program, 6 × 90 min group sessions, 6–8 weeks 9 groups with 6 siblings each Parallel parent education component with parent manual 3 month follow-up	Identifying and regulating emotions, problem-solving in challenging situations, knowledge of NDC family information exchange, connecting with other siblings	CBCL (parent) SPQ (NAS) (sibling and parent) Sibling Knowledge of CI/DD (structured interview, custom) Sibling Connectedness scale (sibling−dyad relationship, custom) Participant Satisfaction (1–5 scale) (no other intervention evaluation)
McLinden et al. (1991) U.S	N/R All identified as having MR, physical handicap (PH) or ‘multiply handicapped’ (MH; including MR and PH)	6 9.17 (range N/R) 17% M, 83% F 100% Caucasian Siblings’ handicap: 17% MR, 17% PH, 67% MH	5 10.6 (range N/R) 60% M, 40% F 80% Caucasian, 20% Hispanic Siblings’ handicap: 80% MR, 20% MH Not waitlist, different activities	One-hour group session per week for 6 weeks. Sessions held by two state-certified school psychologists. One activity per week (e.g. ‘Dear Aunt Blaby’, role-play, homework, completion of a ‘feelings book’)	Expression of feelings, education on NDCs, coping strategies, self-concept, sibling interactions	PHSCS (self-esteem) Let’s Grow Together (positive feelings or attitudes, custom) Who Helps Me (social support, custom)
Phillips (1999) U.S	All siblings had ‘developmental disabilities’ with mild to moderate MR Further data N/R	90 Overall (*n* = 180): 11.3 (9–12) 72 M (40%), 108F African-American ethnicity, low-income families	90 Overall (*n* = 180): 11.3 (9–12) 72 M (40%), 108F African-American ethnicity, low-income families Waitlist control	RCT of a community-based after-school program. Every weekday for 2.5 h, 15 weeks. Group discussions (40–45 min), homework assistance, and education on NDCs (one topic per week)	Socioemotional functioning, self-esteem, anxiety, depression, chronic stress, family functioning, sibling relationship, and perception of social support	CDI (depression) CMAS-R (anxiety) SEQ (self-esteem) PSSS-R (social support) DHQ (stress) FES (family functioning) SRQ (sibling relationship) No evaluation survey
Roberts et al. (2015) Australia	42 (across control and treatment) 8.86 (4–17) Age difference 0–7 years range All autistic	22 9.3 (7.5–12.5 years) 12 M 10F 48.8% older than disabled sibling	20 Mean age N/R (7.5–12.5 years) 5 M, 15F 50% older Waitlist control	Alternating allocation controlled trial SibworkS: group-based manualised support program, 2 h weekly after school. Treatment had 4 × 6 week groups with 6–8 children each Parent component with parenting manual 3-month follow-up	Cognitive−behavioural therapy principles on goal-setting, sharing knowledge, expressing feeling, seeking social support, coping with stress, and problem-solving	SSSC (social support) SRCS (coping) RSES (self-esteem) ‘What I’ve learned’ Evaluation (knowledge) Satisfaction survey
Smith and Perry (2005) U.K	23 Other data N/R Autism (23), other DD (8)	26 10.63 (6–16 years) 14F, 12 M 53.8% older than disabled sibling	None	Weekly group for 8 weeks. No further information reported	Increasing knowledge, sharing feelings, learning coping skills, enhancing self-concept	PHSCS (self-esteem) Coping and Adjustment Scale (anger/resentment sub-scale) Autism Knowledge Measure for Young Children Intervention evaluation (interview)
Williams et al. (2003) U.S	Treatment: 79 10.8 (1–19 years) Diagnoses (%): CF (5.1), diabetes (27.8), spina bifida (12.7), cancer (7.6), DDs (46.8) Control: 102 9 (1–19 years) Diagnoses (%) CF (3.9), diabetes (43.1), spina bifida (5.9), cancer (6.9), DDs (40.2)	79 (full) 71 (partial) Full: 79 11.1 (7–16 years) 51.9%M 87.3% white ethnicity Partial: data N/R	102 11.2 (6–16 years) 54.9%M 86.1% white ethnicity Waitlist control	Randomised, 3 groups (full, partial intervention and waitlist control), Full intervention: structured teaching about NDCs, psychosocial sessions, a 5-day residential summer camp, and two booster sibling sessions. Included parent education component. Partial treatment = camp only condition Assessments: baseline and 5 days post-intervention, 4, 9, 12 months	Sibling knowledge, sibling social support, self-esteem, mood and positive affect, behaviour problems, sibling attitude towards illness/NDC	SSSC (social support) SPPC (self-esteem) SPQ (sub-scale mood) SPQ (NAS) No evaluation survey
Zucker et al. (2021) (same participant data as Jones et al., 2020)	44 7.43(3.37) (3–17) All autistic Support group: 3F, 18 M Control group: 5F, 18 M	24 8.31(3.52) (3.5–18) 10F, 14 M 40% older than disabled sibling 4 white, 3 mixed race, 13 Asian, 2 Hispanic, 1 Guyanese	30 8.62(3.75) (4–15) 20F, 10 M 50% older than disabled sibling 6 white, 6 mixed race, 10 Asian, 6 Hispanic, 1 Guyanese Attention-only condition	Secondary data analysis on Jones et al. (2020) Randomised controlled trial over 10 weeks with an attention-only control. Lessons focussed on psycho-education, discussing emotions, learning coping skills, knowledge of autism, support network	Sibling−dyad relationship quality improvement	Observational coding during free play sessions in 2 × 5 min blocks by group facilitators Sibling Relationship Questionnaire (SIB-S) (modified Buhrmester & Furman, 1990) SIB-P (parent-report on sibling relationship) CBCL (parent)
Mixed methods studies						
D’Arcy et al. (2005) U.K	Data N/R All had ‘physical or intellectual disability or both’	16 8–10 years 11 M, 5F	None	SibShops model, monthly × 4 months, Saturday 10am–1 pm, psycho-education and recreation Parent and professional education opportunities in parallel Pre/post-intervention only	Discuss experiences, learn coping skills, learn about NDC, share common joys and worries	PHSCS (self-esteem) Sibling interview at home No evaluation survey
Fell et al. (2022) U.S	44 6–17 years All autistic	37 15(1.2), 14–17 years 53% F (4 intervention groups of 8–10 participants) 80% white, 11% Hispanic/Latino	None	Modified Stress Management and Resiliency Training-Relaxation Response Resiliency Program [SMART-3RP “SibChat” 1 h virtual sessions weekly over 8 weeks. Incorporating CBT and positive psychology. Run by two clinical psychoogists. Program piloted on a sibling advisory panel	Improve sibling resiliency and stress coping abilities through mind–body techniques to elicit relaxation response, promote positivity, and empowering states of mind	Quantitative survey on benefits of intervention (non-standardised) Qualitative sibling interview
Hayden et al. (2019) U.K	Data N/R 49.1% autism, 51.9% included DS and other ‘chronic conditions’	55 (from 11 schools) 9.18 (7–11) 54.5% F 43.6% white-British, 23.6% Pakistani	None	Sibs Talk. One-to-one manualised support for school students. 10 sessions of 25–35 min over 1 school term. Focus on sibling’s feelings, experiences, challenges Pre/post-intervention only	Improve overall wellbeing, engagement with learning. Reduce anxiety	HIFAMS.(positive mood, social support) SDQ (parent) Evaluation interview
Qualitative studies						
Calio and Higgins-D’Alessandro (2021) U.S	6 19.5 (11–26 years) Autism All male 5 living at home with parents, 1 living in a group home	6 21 (20–22 years) 4 M, 2F Two older than the autistic sibling All Caucasian university students, Catholic or Christian upbringing	None	Peer-led structured support group for university students; the ‘Sibling Allies’ model. 5 sessions, 35–65 min every second week. Topics: sense of responsibility, leaving home, misconceptions, community interactions, maturity, family relationships, school experience, worries about the future. Follow-up survey 1–2 years post-intervention	Evaluate group’s meaningfulness and usefulness; improve sibling willingness to share experiences; improve social connections	Thematic analysis conducted by study authors of 5 recorded support group discussions, using Multi-Grounded Theory (MGT) to develop causal relationships between conditions (contexts, background), actions (response to NDC), and consequences (attitudes, feelings, and resulting circumstances)
Fjermestad et al. (2019) Norway	Data N/R NDCs (*n*) Rare chromosomal conditions with ID (16), spinal muscular atrophy (7), Sotos syndrome (6), Friedreich’s ataxia (5), 22q11.2 deletion syndrome (5), neuronal ceroid lipofuscinoses (2), ID (2), other (5)	58 11.4 (7–17 years) 69%F 55.2% older than disabled sibling All Caucasian	None	Age-matched groups of 4–7 children, met for 3 × 60 min sessions over 5-day residential stay; 1 group leader and 1 facilitator. Topics on: diagnostic information, creative art, emotions and coping	Knowledge of NDC, emotion regulation and expression, family communication	Video recordings of 20 regular group sessions with 11 groups (verbal statements coded)
McCullough and Simon (2011) U.K	3 All male All autistic with ID and limited verbal ability	3 1F age 7, 1 M age 8, 1F age 10. (Broader group, but these 3 participants most regular attendees)	None	Psycho-educational strengths-based group intervention Strengths-based activities to highlight special traits of siblings, build positive feelings, self-efficacy, and self-esteem	Socialising techniques, affective expression, overcome isolation, better communication with family	Feasibility and acceptance evaluation Themes identified through behavioural observation and conversation (No interview protocol)
Naylor and Prescott (2004) Canada	55 N/R All LD	55 8–18 years	None	Group-based support and discussion Quality of life approach to foster support, safeguarding, autonomy and resilience	Feasibility study to establish needs of siblings, reflect on direct experiences and feelings of siblings	Evaluation and feasibility through dialogue with siblings Final evaluation interview to highlight changes in siblings’ perceptions (No interview protocol)
Rye et al. (2018) U.K	All under 16 years Other data N/R All had moderate to severe NDC (autism, severe LD, chromosomal deletion, CP, epilepsy)	7 8–13 years 1 M, 6F (the 1 M stopped after one session) 2 White-British, 1 Asian British, 1 mixed race British	None	Community intervention offered through child and adolescent mental health service. Based on Sibs (UK) manualised program. 10 weekly 2-h sessions after school adopting a strengths-based approach	Improve family communication, problem-solving coping strategies, increase knowledge of NDC, recognise and describe feelings	Semi-structured interview evaluation during final session (n = 4 in attendance) with thematic analysis

Diagnosis abbreviations: *Autism* autism spectrum conditions (including studies using the term ‘ASD’ for autism spectrum disorder and previously used diagnostic terms Asperger’s syndrome and PDD-NOS); *ADHD* attention-deficit hyperactive disorder; *CF* cystic fibrosis; *CP* cerebral palsy; *DD* developmental disorder; *DS* Down syndrome; *ID* intellectual disability; *LD* learning disability; *MR* mental retardation; *NDC* neurodevelopmental condition; *WS* Williams Syndrome

Instrument abbreviations: *SDQ* Strengths and Difficulties Questionnaire; *CDI* Children’s Depression Inventory-Short Form (CDI-S); *CCSC* Children’s Coping Strategies; *CMAS-R* Children’s Manifest Anxiety Scale-Revised; *DASS* Depression and Anxiety Stress Scales; *DHQ* Daily Hassles Questionnaire; *FES* Family Environment Scale; *HIFAMS* ‘How I Feel About My School’ Questionnaire; *PedsQL 4.0* Paediatric Quality of Life Inventory™ Version 4.0; *PHSCS* Piers-Harris Children’s Self-Concept Scale; *PCCS* Parent–Child Communication Scale; *PSSS-R* Perceived Social Support Scale-Revised; *RCMAS* Revised Children’s Manifest Anxiety Scale; *RSES* Rosenberg Self-Esteem Scale; *SEQ* Self-Esteem Questionnaire; *SPQ* Siblings Problems Questionnaire; *SPPC* Self-Perception Profile for Children; *SRCS* Self-Report Coping Scale; *SRQ* Sibling Relationships Questionnaire; *SSSC* Social Support Scale for Children

**Table 3:** Results of individual studies

Study	Outcome	Retention rates	Results (within- and between-group comparisons)	Direction of effect, size*	Study limitations
Quantitative					
Brouzos et al. (2017) Greece	Emotional adjustment Coping Knowledge	N/R	Intervention group significantly improved on all measures Group comparisons: significantly favoured intervention group SDQ pre–post-intervention *t*(21) = 7.67, *p* < .001 vs control group *t*(15) = 2.08, *p* = .05. *F*(1, 36) = 168.38, *p* < .001, *η*^2^ = .82. *F*(1, 36) = 46.40, *p* < .001, partial *η*^2^ = .56 Coping/adjustment: intervention, *t*(21) = 16.05, *p* < .001 vs control group *t*(15) = .55, *p* = .59 The Knowledge of Autism Syndrome, intervention group *t*(21) = 11.42, *p* < .001, vs control group *t*(15) = 1.96, *p* = .07. *F*(1, 36) = 70.68, *p* < .001, *η*^2^ = .66	*Pre–post* Positive, large *Group* Positive, large	Autistic symptom severity not measured No ongoing evaluations or follow-up Small sample size Diagnoses from DSM-IV (changes in DSM-5 for autism) Self-report only, not multi-informant Only test–retest control group (not alternative active intervention)
Evans et al. (2001) U.K.	Self-esteem Knowledge	N/R	Increase in self-esteem post-intervention: *t*(14) = 2.18, *p* < .05, pre: 35.53, post 32.29 Knowledge questionnaire: all participants improved (data N/R) Evaluation: participants reported positive experiences (outdoor activities, meeting other children, making masks or puppets, completing a ‘feelings’ chart’)	*Pre**–**post* Positive, small	Small sample Relied on parental feedback for intervention evaluations
Fjermestad et al. (2020) Cambodia	Depression Emotional adjustment Family wellbeing	At 4 month follow-up, data available from 74.1% of participating siblings	Sibling self-report SDQ better than parent−report SDQ at baseline No significant differences post-intervention on SDQ (sibling) or parent–child communication (sibling and parent report) Parent self-reported mental health (DASS; *d* = 0.44) and parent-reported sibling mental health (SDQ; *d* = 0.52) significantly improved at 4 month follow-up Evaluation survey: mean score 3.4 (range 1–4)	*Pre**–**post* Neutral	No control group Outcomes measured 4 months after (not immediately post-intervention) 25% of sample lost to follow-up Heterogeneous NDCs, limits generalisability. Unknown cultural confounders in Cambodia (program piloted in Norway)
Gettings et al. (2015) U.K.	Quality of life Emotional adjustment	2 siblings stopped after one session 3 occasions of non-attendance out of a possible 48 episodes (93%)	SDQ scores worsened (more difficulties) post-intervention (non-significant). QOL scores on PedsQL 4.0 worsened (poorer QOL) for five siblings (data N/R) SVQ pre-intervention: emotional responses included “fear, anger, upset, feeling hurt, sense of injustice, worry, shock.” SVQ post-intervention: all had reduction in severity of > 1 concern, 3 reported > 1 concern worsened due to worsening symptoms (e.g. negative mood) Evaluation: all siblings experienced benefits, e.g. reduced isolation, building friendships and talking openly. “I’ve learnt I’m not alone”, “chance to talk about problems freely”	*Pre**–**post* Negative, small	Small sample size Challenges for children with hearing or speaking difficulties to use the audioconference method Convenience sample No control group
Giallo and Gavidia-Payne (2008) Australia	Stress Coping	1 family in the waitlist dropped out due to limited availability; 2 families completed the intervention but did not return post-group surveys. No significant differences between groups at baseline	Sibling report pre–post: lower perceived intensity (but not frequency) daily hassles and stress (*p* = 0.022, *η*^2^ = 0.26) Less use of distancing as coping (*p* = .006, *η*^2^ = 0.35) Evaluation: 78% strongly agreed the skills learnt were appropriate and useful; helped them deal with stress (83.3%) and better cope with problems (88.9%). Parent evaluations: 84–100% positive ratings, improved their parenting skills and family communication Parent report: SDQ intervention group improved compared to waitlist group, *F*(1,18) = 4.86, *p* = .041, *η*^2^ = .10. Parent−report SDQ in the waitlist group worsened. Intervention group: 10 of 12 siblings had ‘clinically reliable’ improvements (parent report on Reliable Change Index)	*Pre–post* Positive, small *Group* Positive, small	Small sample size Younger children understood less intervention content Could not determine which intervention components were most effective 6 weeks may be too short to observe significant changes No follow-up data collected
Haukeland et al. (2020) Norway	Emotional adjustment Family wellbeing Knowledge	*T*1 measures completed *n* = 107; received intervention *n* = 99; *T*2 post-intervention completed *n* = 77 (response rate 88.8%, 18.4 weeks after *T*1); follow-up *T*3 *n* = 55 completed (response rate 55.6%, 33.4 weeks after *T*1) Group leaders’ adherence to manual, average fidelity 85.6% (range 78.7–93.9%)	Baseline parent and sibling report SDQ within upper limits of normative range SDQ (sibling): problems decreased significantly over time (*p* = 0.009); *d* (*T*2; *T*1) = 0.23, *d* (*T*3; *T*1) = 0.31 Improved sibling-rated communication on PCCSc over time (*b* = 0.14, *p* = 0.001); *d* (*T*2;*T*1) = 0.26, *d* (*T*3; *T*1) = 0.48 CD-knowledge (SKI): significant improvement over time (*p* < 0.001); *d* (*T*2;*T*1) = 0.37, *d* (*T*3;*T*1) = 0.64 NAS: significant decrease in scores over time (*p* = 0.003); *d* (*T*2; *T*1) = 0.22, *d* (*T*3; *T*1) = 0.26 Evaluation M(SD) on overall satisfaction with intervention 3.5(0.6) (range 2–4); M(SD) for perceived importance of the intervention amongst siblings 8.1(1.9) and for perceived utility 8.2(1.8) Parent−report SDQ improved over time; significant for father report, not maternal report: SDQ *T*1–*T*2: mother (*d* = 0.09), father (*d* = 0.10); *T*2–*T*3: mother (*d* = 0.21), father (*d* = 0.24)	*Pre–post* Positive, medium	Not a controlled design Heterogeneous NDCs, did not assess symptom severity Sibling had wide age range All parents high SES Conducted across various settings (variable timeframe, staff, location, number of participants and leisure content)
Jones et al. (2020) U.S.	Anxiety Depression Social wellbeing Coping	34 allocated to attention group (30 completed, 88%), 30 allocated to support group (24 completed, 80%) Group leader fidelity 71–100%. 88% of intervention group and 96% control group attended 7 + sessions	Non-significant changes: decreased depressive symptoms, increased coping skills, increased anxiety for both groups Autistic sibling’s autism severity score significantly moderated relationship between group and post-intervention depression scores (*β* = 1.40, *t* = 3.28, *p* = .003); and anxiety scores (*β* = .74, *t* = 2.21, *p* = .04) Coping: intervention group improved pre–post (*p* = .01); control group no change (*p* = .50). At baseline intervention group had significantly lower coping skills than control group (*p* = .02), no group differences post (*p* = .15) Parent−report CBCL siblings in the intervention group had significant improvements in externalising behaviour and coping skills, compared to control group	*Pre–post* Positive, small *Group* Neutral	Underpowered to examine subgroups of siblings Could not adjust for gender of either sibling, birth order or behaviour problems in the autistic child Required more demographically homogeneous sibling groups (e.g. closer in age)
Kang et al. (2021) South Korea	Stress Self-esteem	33 participants randomized; 4 dropped out due to personal reasons	Stress scale: intervention group significantly improved pre–post on all sub-scales: parents (*t* = 2.226, *p* < 0.05), family (*t* = 2.941, *p* < 0.05), friends (*t* = 2.460, *p* < 0.05), study (*t* = 2.609, *p* < 0.05) and school (*t* = 2.676, *p* < 0.05) Self-esteem: significant improvement in overall self-esteem and social self-esteem (*t* = − 5.140, *p* < 0.05 and *t* = − 2.629, *p* < 0.05); not self-esteem at home and school (*t* = − 1.967, *p* < 0.05 and *t* = 0.558, *p* < 0.05) Control group had no significant changes on any measures (N/R)	*Pre–post* Positive, small *Group* Positive, small	Small sample size High dropout rate in control group, impairs generalisation. Children had to be accompanied by parents, limited recruitment No measures of family demographics/function Limit to regional generalisation Underpowered (required *n* = 33)
Kryzak et al. (2014) U.S.	Anxiety Depression Knowledge	Retention rate N/R. Did not measure fidelity to manualised intervention	Earlier pilot of Jones et al. (2020) intervention CDI significant pre–post decrease on total CDI and negative self-esteem subscale scores *t*(9) = 2.533, *p* = 0.032 and *t*(9) = 2.540, *p* = 0.032 RCMAS-2 pre–post-test decreased for all sub-scales, only significant for physiological anxiety, *t*(7) = 2.886, *p* = 0.023 Sibling knowledge pre–post: % of correct responses on knowledge increased, *t*(10) = − 1.901, *p* = 0.086 Social network questions increased significantly pre–post: *t*(10) = − 3.525, *p* = 0.005 Sibling evaluation: high satisfaction M(SD) 4.56(0.67); self-reported positive outcomes at program end M(SD) 4.09(1.13)	*Pre–post* Positive, small	Autistic symptom severity not measured No measures of coping. Underpowered sample size Did not directly measure treatment fidelity No control group Siblings had varying baseline functioning (although most not in clinical range)
Lobato and Kao (2002) U.S.	Emotional adjustment Family wellbeing Knowledge	75% of families attended all six sessions; all 54 siblings completed pre–post data. Follow-up at 3 months *n* = 20 (37%)	Sibling self-reported significant decreases on SPQ NAS pre–post (*F*(1, 53) = 4.58, *p* < .05), not maintained at 3 months post-treatment. Sibling report M(D): full sample (*n* = 54) pre vs post: 2.3 (0.42) vs 2.2 (0.47), *p* < .05. Follow-up sample (*n* = 20): pre vs post vs 3 month follow-up: 2.2 (0.46) vs 2.2 (0.51) vs 2.1 (0.39) (n.s.) Sibling connectedness increased pre to post: *F*(1, 53) = 44.20, *p* < .01; and at 3 months: *F*(2, 18) = 9.20, *p* < .01 Sibling ability to accurately explain disability, *F*(2, 18) = 3.60, *p* < .05. Scores increased pre–post (*p* < .05) but decreased at 3 month follow-up (*p* < .05) Parent report on SPQ (NAS) and CBCL: NAS results non-significant post-treatment: *F*(1, 43) = 2.60, *p* < .11; significant decreases in CBCL internalizing, *F*(1, 39) = 9.41, *p* < .01 and externalizing, *F*(1, 39) = 13.40, *p* < .01 post treatment CBCL externalising scores continued to decrease at 3 months (*p* < .05), internalising scores did not (*p* < .09) At 3-months (*n* = 20), sibling connectedness increased significantly from baseline (parents, *F*(2, 14) = 7.39, *p* < .01)	*Pre–post* Positive, small	No control group Not designed to test interacting sibling and parent components Demographics favoured Caucasian, middle-class, 2-parent families Heterogeneous NDCs (interaction effects found based on diagnosis)
McLinden et al. (1991) U.S.	Self-esteem Social wellbeing	N/R	Scores of both groups at pre-test within normal range Significant post-intervention group difference in total score of Who Helps Me favouring intervention group PHCSCS intervention v control: *F* value 1.78, *p* = 0.22 Who Helps Me: intervention v control: *F* value 5.85, *p* = 0.04 Let’s Grow Together: intervention v control: *F* value 0.00, *p* = 0.98	*Pre–post* Neutral, one significant improvement *Group* Neutral, one significant group difference	No psychometric data or norms for two instruments Small sample size Sampling bias. Over-representation of female participants No evaluation survey to examine perceived beneficial components
Phillips (1999) U.S.	Anxiety Depression Stress Self-esteem Social wellbeing Family wellbeing	N/R	Intervention group: decreased anxiety and depression, less sibling-related stress, improved self-esteem Control group: no significant change PSSS-R (social support): significant differences for peers, school personnel, and community-center staff subscales (intervention group only) FES (family functioning) and sibling relationship did not show significant intervention effects (data N/R) Data at Time 2 vs Time 1, group comparison (all favouring intervention group post-intervention) Depression: *F* = 4.33, *p* < .05; Anxiety: *F* = 4.31, *p* < .05; Self-esteem/peers: *F* = 5.47, *p* < .05; Self-esteem/school: *F* = 5.45, *p* < .01; Self-esteem/family: *F* = 4.36, *p* < .05; Self-esteem/global: *F* = 4.32, *p* < .01; Daily hassles/siblings: *F* = 4.77, *p* < .01; Social support/peers: *F* = 4.45, *p* < .01 Social support/school: *F* = 4.23, *p* < .01; Social support/center staff: *F* = 4.33, *p* < .01	*Pre–post* Positive, moderate *Group* Positive, small	Could not determine which intervention components were most effective Underpowered to detect large effect sizes Generalisable only to African American low-income population
Roberts et al. (2015) Australia	Self-esteem Social wellbeing Coping	30 allocated to treatment, 26 allocated to waitlist control. Follow-up data available: treatment group *n* = 14 (63.6%), control group *n* = 16 (80%) Attended average 5.4 of 6 sessions Fidelity reported by group facilitators: on average 94% of activities completed across six sessions	At baseline 60% total participants ‘borderline’ or ‘abnormal’ on parent-reported SDQ Post-intervention, treatment group reported less coping through externalising, improved self-esteem (medium effect sizes), not statistically significant and not maintained at 3 month follow-up Group × time effects at post-test and 3-month follow-up: Coping: Social support: *F*(1,37) = 1.16, *p* = .29, *η*^2^ = .03, follow-up *F*(1,27) = 1.35, *p* = .26, *η*^2^ = .05 Coping: Problem solving: post *F*(1,37) < 1, follow-up *F*(1,27) = 1.06, *p* = .31, *η*^2^ = .04 Coping: Distancing: post *F*(1,37) < 1, follow-up *F*(1,27) = 1.08, *p* = .31, *η*^2^ = .0 Coping: Externalising: post *F*(1,37) = 2.88, *p* = .10, *η*^2^ = 07, follow-up *F*(1,27) < 1 Self-esteem: post *F*(1,37) = 2.23, *p* = .14, *η*^2^ = .06, follow-up *F*(1,29) < 1 Qualitative analysis: social support from peers from the group, coping and problem-solving Evaluation: 90% siblings rated program helpful Parent-reported SDQ at post-treatment and 3 month follow-up had significant positive large effect sizes favouring the treatment group: post: *F*(1,36) = 4.92, *p* = .03, *η*^2^ = .12; 3 month: *F*(1,30) = 6.20, *p* = .02, *η*^2^ = .17 (no sibling self-report SDQ)	*Pre–post* Positive, small *Group* Neutral	Measurement issues (parent-reported child functioning displayed treatment effects) Siblings may have under-reported baseline difficulties Parents aware of program aims (reporting bias risk) Lack of accessibility to families. Underpowered to detect medium effect sizes More participants in treatment group than control had participated in another support group within 6 weeks prior to intervention (41% vs 15%, *p* = 0.03)
Smith and Perry (2005) U.K.	Self-esteem Coping Knowledge	5 did not complete the intervention (83.9% retention). 7 of 26 completers participated more than once; repeaters and single-attenders did not differ significantly at baseline	Significant increase in self-concept on the PHSCS post-test. Significant improvement on knowledge. No significant difference on coping/adjustment 7 repeaters exhibited additive beneficial effects (repeat dates or time intervals N/R) Outcomes M(SD) at pre-test vs post-test: Piers-Harris: *t*(25) = − 2.84, *p* = .005 Knowledge: *t*(24) = − 2.45, *p* = .01 Coping: *t*(25) = .95, *p* = .18 Qualitative evaluation: positive overall Parent−report CBCL at baseline only, borderline-clinical range: 9 siblings (36%) internalizing problems; 5 (20%) externalising problems; 4 (16%) both	*Pre–post* Positive (additive effect), small	No control group No long-term follow-up
Williams et al. (2003)	Self-esteem Emotional adjustment Social wellbeing Family wellbeing	Initial withdrawal rates 14% in all groups 252 of 292 completed all follow-up data collection points. 7 of the 40 withdrawals occurred because parent could not attend all 5 data collection points	19 of the 23 tests showed significant improvements from baseline to immediately post-treatment (*p* < .05) in full intervention group. In partial group, improvements on 11 of 23. In control group, improvements on 9 of 23 Sibling self-Esteem: full and partial treatment groups statistically significant sustained gains; > 5.5 points (approx. 5.0% gain) Sibling mood: full treatment group statistically significant gains 9 and 12 months after baseline; > 2.5 points (approx. 5.0% gain) Sibling behaviour problems: full treatment group statistically significant (*p* < .01) declines in problems; > 5 points; 9 and 12 months after baseline (approx. 5.5% gain) Sibling negative attitudes: significant improvements in treatment and control groups, largest gains in full treatment group > 8 points (approx. 25% gain) Teacher and parent report Eyberg Child Behaviour Inventory: full treatment group significant improvements 9 and 12 months after baseline (> 5 points, approx. 5.5% gain)	*Pre–post* Positive (dose–response), large *Group* Positive, small (group: full > partial > control)	Cost of attending the program (US$450–500 per parent–child dyad) Cannot generalised to low-income SES minority populations Raw data not provided for analysis of effect sizes
Zucker et al. (2021)	Family wellbeing	See Jones et al. (2020) above	Main effect of group (support vs control) statistically significant for sibling relationship parent report (*p* < .01) Main effect of time (pre–post intervention) statistically significant for sibling relationship parent report (*p* < .05) Sibling relationship increased over time for support group, decreased for control group; significant group × time interaction (*p* < .01) Effect sizes ranged *η*^2^ = 0.078–0.203 Intervention group SIB-S M(SD) pre 73.62(14.78) v post 84.27(7.48) Control group SIB-S M(SD) pre 79.24(17.78) v post 75.83(16.95) Time effect for intervention group: *F*(1, 18) = 16.1, *p* < 0.001; *ηp*^2^ = 0.179) Group effect post-intervention, significantly higher for support group: *F*(1, 41) = 4.06, *p* = 0.05; *ηp*^2^ = 0.090 Group × time interaction: *F*(1,41) = 10.45, *p* = 0.002, *ηp*^2^ = 203	*Pre–post* Positive, small *Group* Positive, small	Discrepancy in parent report. Baseline data only reported on 3–4 weeks prior Did not obtain autistic child’s perceptive Excluded siblings of autistic children with severe problematic behaviour Could not analyse sub-groups on age, gender or birth order No BAP data collected
Mixed methods					
D’Arcy et al. (2005) U.K.	Self-esteem Coping Knowledge	N/R	No significant improvement on self-esteem scores, some siblings had lower (worse self-esteem) mean scores post-intervention; 2 siblings had clinically low self-esteem post-intervention PHSCSC total and subscale scores pre–post: Total: *t*(14) = − 1.064, *p* = .305 Behaviour: *t*(14) = − 1.351, *p* = 0.198 Intellectual/school status: *t*(14) = − 0.381, *p* = 0.709 Physical appearance/attributes: *t*(14) = 1.128, *p* = 0.278 Anxiety: *t*(14) = − 1.763, *p* = 0.100 Popularity: *t*(14) = 0.695, *p* = 0.499 Evaluation interview themes: 11 (68.8%) rated workshops ‘excellent’ or ‘very good’ 14/16 said SibShops was enjoyable; 12/16 benefitted from talking about experiences; 8/16 ‘helped with talking about tricky situations’; All siblings improved knowledge of NDC	*Pre–post* Neutral	Small sample size Participants not matched for gender Did not collect data on severity of disability, aspects of family composition or socio-economic status Did not examine factors intrinsic to the child which may act as ‘buffers’ No long-term follow-up
Fell et al. (2022) U.S.	Stress Coping Social wellbeing	40 siblings recruited; 3 withdrew pre-intervention, 2 during intervention (scheduling conflicts *n* = 4, lack of interest *n* = 1)	Quantitative survey: 86% reported intervention had right amount of sessions; 89% indicated sessions were the right length; 74% agreed with the structure; 74% liked group session format, 74% found mind–body relaxation exercises helpful, 71% reported it helped their ability to cope with life stressors. 80% reported practicing relaxation exercises alone a few times per week Qualitative interviews: satisfied with virtual delivery, effective and convenient. Helpful program aspects included: resiliency techniques, making social connections, improvement in sleep, stress, worry and anxiety Siblings’ suggestions for improvement: more group engagement; more ethnic, cultural and geographic diversity Overall helpfulness of sessions score out of 5 = 2 (SD 0.94)	*Pre–post* Positive, small	Did not collect data on severity of NDC or demographics of NDC siblings Sample size lacking in racial and ethnic diversity Recruitment bias due to recruiting via professional parent and sibling organisations Participants were self-selected and may represent siblings with more interest in mind–body therapy
Hayden et al. (2019) U.K.	Quality of life Coping Knowledge	55 evaluation questionnaires from 11 schools were returned (for 8 of these, children who completed the intervention also completed the questionnaire). The remaining three schools each returned four out of five completed questionnaires	HIFAMS changes not statistically significant M(SD): pre 11.31(1.975) vs post 11.37(1.98), *d* = 0.03 (small) Written responses: Learning and Understanding (*n* = 24); Communication and Relationships (*n* = 21); Coping Strategies (*n* = 12) (e.g. ‘I take a deep breath and count to ten and walk away’. ‘when I don’t want to say my feelings out loud I write in my feelings box’); Challenges and Responsibilities (*n* = 16) (e.g. ‘My brother’s behaviour is very violent, [I have learnt] how to deal with tough stuff’) Parent−report SDQ post-intervention significant improvements in hyperactivity scores (*p* < .001, *d* = 0.55), prosocial behaviours (*p* = .002, *d* = 0.47), total SDQ difficulties (*p* = .009, *d* = 0.38) and emotional problems (*p* = .016, *d* = 0.38). (No sibling report SDQ)	*Pre–post* Positive, small	Not a randomised control trial Possible response bias Small sample size Schools were only able to allocate minimal staff to intervention (not representative of the schools generally) Variation in school staff members on knowledge and experience of NDC Limited measures No follow-up questionnaire Sibling feedback based on specific prompts (influenced responses)
Qualitative					
Calio and Higgings-D’Alessandro (2021) U.S.	Coping Social wellbeing Family wellbeing Knowledge	6 university students invited (including the participant-researcher); 6 retained for the study	3 exclusive coded categories: Individual experience of the TDS, Family Life and Family functioning in public All siblings found the group meaningful: themes of Individual Openness and Comfort, the Group’s Shared Emotional Experiences and the Group’s Future Hopes 10 action-paradigm models: responsibility of the TDS, social isolation, TDS’s future, sibling to sibling relationship, ‘finding your normal’, age order of siblings, relationship with parents, family functioning in private, the family’s emotional experience, extended family and family functioning in public with the effects of stigma	*Pre–post* Positive, small	Support group did not aim to provide therapy, only offer siblings the opportunity to share experiences and issues Difficult to evaluate effects on mental health due to format. Small homogeneous group with all male autistic siblings Did not consider other non-disabled siblings in the family Only one group facilitator, also a participant and study author
Fjermestad et al. (2019) Norway	Coping	80 (77%) of 105 approached families consented to participate. Qualitative data available for 72.5% of the total sample (i.e. 58 of 80)	No pre/post measures or intervention evaluation Thematic analysis conducted by study authors of group discussion transcripts resulted in six main wellbeing themes: (1) Abilities (physical, cognitive, social), (2) Intentions (understanding the intentions of their siblings), (3) Insight (knowledge of sibling), (4) Emotions (described vs explained; explicit vs implicit; constant vs changing; present vs predicted; observed vs inferred; genuine vs fake), (5) Personality, preferences and desires (learning expressions of needs and coping skills) and (6) Normality versus difference (acceptance and knowledge)	*Pre–post* Positive, small	Study design may have limited group discussions Heterogeneous group of rare NDCs limit generalisability Group participation (not individual discussion) may have limited openness Parents from a higher education than Norwegian average No SES or ethnic diversity Only post-intervention discussion without baseline testing
McCullough and Simon (2011) U.K.	Self-esteem Social wellbeing Family wellbeing Quality of life	N/R	Key themes pre-intervention: sense of isolation, lack of belongingness and uncertain identities Post-intervention: all siblings had found positive qualities about themselves to improve self-perception, better QOL Thematic analysis of interviews citing benefits of the program: improved social skills, decreased self-alienation, increased parent–child communication, more conversations with families, improved inner value and self-worth (e.g. “stronger person”, a “good person”)	*Pre–post* Positive, small	No conclusions on intervention effects due to qualitative process Small sample size Recruitment problems (parents did not support their children attending)
Naylor and Prescott (2004) Canada	Depression Self-esteem Social wellbeing Quality of life	N/R	Thematic analysis of experiences of the support group: net positive effect on sibling mental health and wellbeing and QOL Evaluation: all siblings enjoyed it; helped them make new friends; increased self-esteem, ‘it has helped me be more patient’, better coping strategies (‘she doesn’t annoy me as much’) but still had depressive symptoms (‘it gets me down’, ‘I feel left out’). Some reflections were negative on sibling mental health, e.g. siblings feel ‘isolated,’, ‘not have enough parents’ attention’	*Pre–post* Neutral	No conclusions on intervention effects due to qualitative process No pre–post intervention quantitative data available Small sample size
Rye et al. (2018)	Self-esteem Social wellbeing Knowledge Coping	10 invited. 7 attended first session. 4 completed final session and interview evaluation (40% retention)	Thematic analysis of interviews, 5 themes with supporting quotes: (a) Meeting similar people (“it makes me feel less sad… it is not just you. You are not alone”), (b) A break from home (“forgetting about all your worries and stuff”), (c) Enjoyment of the activities-led group (“I could make friends and play games and stuff”), (d) Building confidence and self-esteem (“it improved my confidence”; “it made me feel a bit more special”, one participant drew a lion and described the group as “brave, powerful and strong”, “because you have to be brave to get through hard times”), (e) Learning and applying knowledge about disabilities (“I learned how to like, how to handle your brother”)	*Pre–post* Positive, moderate	Sample size Low retention rate low (due to transport issues), Interviewer perceived content limited due to participants’ shyness Did not evaluate effects of program on other family members Heterogeneous NDC group

*Direction of effect and size relates to pre–post changes (positive, negative or overall neutral on balance of all measures) for the intervention group and where applicable group differences post-intervention between intervention and control group (where positive indicates the intervention group significantly improved post-intervention compared to control group scores)

**Table 4:** MMAT Quality appraisal of included studies

Study	MMAT quality assessment																						Overall
	Screening		Qualitative					Quantitative RCT					Quantitative NRT					Mixed methods					
	S1	S2	1.1	1.2	1.3	1.4	1.5	2.1	2.2	2.3	2.4	2.5	3.1	3.2	3.3	3.4	3.5	5.1	5.2	5.3	5.4	5.5	*N* (%)
Giallo and Gavidia-Payne (2008)	Y	Y						Y	Y	Y	N	N											3 (60)
Jones et al. (2020)	Y	Y						Y	N	Y	N	Y											3 (60)
Kang et al. (2021)	Y	Y						N	Y	Y	N	Y											3 (60)
Roberts et al. (2015)	Y	Y						N	Y	Y	N	Y											3 (60)
Zucker et al. (2021)	Y	Y						Y	N	Y	N	Y											3 (60)
Williams et al. (2003)	Y	Y						N	Y	N	N	Y											2 (40)
Brouzos et al. (2017)	Y	Y											Y	Y	N	Y	Y						4 (80)
Fjermestad et al. (2020)	Y	Y											Y	Y	Y	N	Y						4 (80)
Haukeland et al. (2020)	Y	Y											Y	Y	Y	N	Y						4 (80)
Lobato and Kao (2002)	Y	Y											Y	Y	Y	N	Y						4 (80)
Phillips (1999)	Y	Y											Y	Y	Y	N	Y						4 (80)
Kryzak et al. (2014)	Y	N											Y	Y	N	N	Y						3 (60)
McLinden et al. (1991)	Y	Y											Y	Y	N	N	Y						3 (60)
Smith and Perry (2005)	Y	Y											Y	N	Y	N	Y						3 (60)
Evans et al. (2001)	Y	Y											N	N	N	N	Y						1 (20)
Gettings et al. (2015)	Y	N											N	N	N	N	Y						1 (20)
D’Arcy et al. (2005)	N	N																Y	Y	N	Y	N	3 (60)
Hayden et al. (2019)	Y	N																Y	N	N	N	Y	2 (40)
Fell et al. (2022)																		Y	N	N	N	N	1 (20)
Calio and Higgins-D’Alessandro (2021)	Y	Y	Y	Y	Y	Y	N																4 (80)
Fjermestad et al. (2019)	Y	Y	Y	Y	Y	Y	N																4 (80)
Rye et al. (2018)	Y	N	Y	Y	Y	N	Y																4 (80)
McCullouh and Simon (2011)	N	N	Y	N	Y	N	N																2 (40)
Naylor and Prescott (2004)	N	N	Y	N	Y	N	N																2 (40)

Screening scores are not included in the total quality score

*MMAT* Mixed Methods Appraisal Tool; *RCT* Randomised Controlled Trial; *NRT* Non-randomised Trial; *S1* Are there clear research questions? *S2* Do the collected data allow to address the research questions?

1.1—Is the qualitative approach appropriate to answer the research question? 1.2—Are the qualitative data collection methods adequate to address the research question? 1.3—Are the findings adequately derived from the data? 1.4—Is the interpretation of results sufficiently substantiated by data? 1.5—Is there coherence between qualitative data sources, collection, analysis and interpretation? 2.1. Is randomization appropriately performed? 2.2. Are the groups comparable at baseline? 2.3. Are there complete outcome data? 2.4. Are outcome assessors blinded to the intervention provided? 2.5 Did the participants adhere to the assigned intervention? 3.1—Are the participants representative of the target population? 3.2—Are measurements appropriate regarding both the outcome and exposure/intervention? 3.3—Are there complete outcome data? 3.4—Are the confounders accounted for in the design and analysis? 3.5—During the study period, is the intervention/exposure administered as intended? 5.1—Is there an adequate rationale for using a mixed methods design to address the research question? 5.2—Are the different components of the study effectively integrated to answer the research question? 5.3—Are the results adequately brought together into overall interpretations? 5.4—Are divergences and inconsistencies between quantitative and qualitative results adequately addressed? 5.5—Do the different components of the study adhere to the quality criteria of each tradition of the methods involved?], *Y* Criteria satisfied, *N* Criteria not satisfied

**Table 5:** Analytical themes sub-themes

Overarching analytical theme (no. of studies)	Sub-themes
Emotional and behavioural adjustment (*k* = 14)	Internalising symptoms
	Externalising symptoms
	Overall mood/affect changes
	Emotion regulation
	Behavioural changes around disabled sibling
Support and connection (*k* = 13)	Connecting with similar siblings
	Learning to ask for help/support
	Sharing similar/different situations
	Maintaining new friendships/support sources
	Community and recreation involvement
Knowledge and education (*k* = 14)	Knowledge of NDC
	Parent−education component
	Learning/sharing coping skills
	Problem-solving techniques
	Understanding and accepting feelings
Inter-personal wellbeing (*k* = 14)	Sibling−dyad relationship
	Sibling peer relationships
	School support and teacher relationships
	Parent–child communication
	Family communication and cohesion
Intra-personal wellbeing (*k* = 19)	Self-worth/self-esteem
	Sense of identity, resilience and bravery
	Achieving autonomy
	Quality of life and happiness
	Internal management of feelings/emotions

**Table 6:** Grade certainty of evidence in outcomes results

Intervention target	Outcome results (no. studies)	No. of participants (no. studies)		Quality of the evidence (GRADE)	Studies (direction of effect)
		Intervention	Comparison		
Anxiety	Improved sig (2) No change (1) Declined non-sig (1)	210 (4)	120 (2)	Low^ab^	Improved: Kryzak et al. (2014), Phillips (1999) No change: Fjermestad et al. (2020) Decline: Jones et al. (2020)
Depression	Improved sig (3) Improved non-sig (1) No change (2)	272 (6)	120 (2)	Low^ab^	Improved: Kryzak et al. (2014), Phillips (1999), Rye et al. (2018) Non-sig imp: Jones et al. (2020) No change: Fjermestad et al. (2020), Naylor and Prescott (2004)
Stress	Improved sig (4)	157 (4)	110 (3)	Low^acd^	Improved: Fell et al. (2022), Giallo and Gavidia-Payne (2008), Kang et al. (2021), Phillips (1999)
Self-esteem	Improved sig (6) Improved non-sig (2) No change (2)	353 (10)	228 (5)	High^b^	Improved: Evans et al. (2001), Kang et al. (2021), Phillips (1999), Williams et al. (2003) Non-sig imp: Roberts et al. (2015), Smith and Perry (2005) No change: D’Arcy et al. (2005), McLinden et al. (1991)
Quality of life	Improved sig (1) Improved non-sig (1) No change (1) Declined (1)	119 (4)	0 (0)	Very low^abcd^	Improved: Hayden et al. (2019) Non-sig imp: McCullough and Simon (2011) No change: Naylor and Prescott (2004) Decline: Gettings et al. (2015)
Emotional adjustment	Improved sig (4) No change (1) Declined (1)	373 (6)	118 (2)	Moderate^cd^	Improved: Brouzos et al. (2017), Haukeland et al. (2020), Lobato and Kao (2002), Williams et al. (2003) No change: Fjermestad et al. (2020) Decline: Gettings et al. (2015)
Social wellbeing	Improved sig (5) Improved non-sig (3) No change (1)	376 (9)	217 (4)	High^d^	Improved: Calio and Higgins-D’Allessandro (2021), McLinden et al. (1991), Phillips (1999), Rye et al. (2018), Williams et al. (2003) Non-sig imp: Fell et al. (2022), McCullough and Simon (2011), Roberts et al. (2015) No change: Naylor and Prescott (2004)
Family wellbeing	Improved sig (6) No change (2)	478 (8)	222 (3)	High^d^	Improved: Calio and Higgins-D’Allessandro (2021), Haukeland et al. (2020), Lobato and Kao (2002), McCullough and Simon (2011), Williams et al. (2003), Zucker et al. (2021) No change: Fjermestad et al. (2020), Phillips (1999)
Coping	Improved sig (6) Improved non-sig (3) No change (1)	298 (10)	75 (4)	Moderate^cd^	Improved: Brouzos et al. (2017), Calio and Higgins-D’Allessandro (2021), D’Arcy et al. (2005), Fjermestad et al. (2019), Giallo and Gavidia-Payne (2008), Hayden et al. (2019) Non-sig imp: Fell et al. (2022), Jones et al. (2020), Roberts et al. (2015) No change: Smith and Perry (2005)
Knowledge	Improved sig (7) Improved non-sig (3)	307 (10)	16 (1)	High^ad^	Improved: Brouzos et al. (2017), Calio and Higgins-D’Allessandro (2021), Evans et al. (2001), Haukeland et al. (2020), Kryzak et al. (2014), Lobato and Kao (2002), Smith and Perry (2005) Non-sig imp: D’Arcy et al. (2005), Hayden et al. (2019), Rye et al. (2018)

GRADE Working Group grades of evidence: High quality: Very confident that the true effect lies close to that of the estimate of the effect. Moderate quality: The true effect is likely to be close to the estimate of the effect, but there is a possibility that it is substantially different. Low quality: The true effect may be substantially different from the estimate of the effect. Very low quality: The true effect is likely to be substantially different from the estimate of effect. Non-sig imp = non-significant improvement reported in results for that outcome (sig = significant)

^a^Imprecision of results (wide confidence intervals, small sample sizes)

^b^Inconsistency (heterogeneity in study results)

^c^Methodology (limitations in study design and implementation and associated risks of bias)

^d^Indirectness of evidence (heterogeneity in measurement tools used or operationalisation of outcome)

## Footnotes

1 We have included in this review neurodevelopmental conditions (NDCs) based on the DSM-5 and DSM-5-TR (text revision, APA, 2022) and the ICD Classification of Mental and Behaviour Disorders, 10th and 11th edition (ICD-10/11; World Health Organisation, 1993). Using these diagnostic criteria, ADHD in organised under the category of an NDC.

2 Although we appreciate and respect that different groups and individuals prefer personalised language conventions, this review adopts person-first language (i.e., person with disability; (Folkins, 1992; Research and Training Center on Independent Living, 2008; Smart, 2001). This decision was made after consultation with sibling consumers and the Sibling Advisory Panel. However, for autism-related discussion, identity-first language will be used (i.e., *autistic sibling*; see Bottema-Beutel et al., 2021). For the purposes of this review, the term *sibling* refers to a sibling of an individual with a neurodevelopmental condition/disability (NDC) or developmental disability; *individual with an NDC* or *disabled sibling* refers to the individual in the sibling dyad who has been diagnosed with an NDC.
